# Smart Gold Nanostructures for Light Mediated Cancer Theranostics: Combining Optical Diagnostics with Photothermal Therapy

**DOI:** 10.1002/advs.201903441

**Published:** 2020-06-18

**Authors:** Tanveer A. Tabish, Priyanka Dey, Sara Mosca, Marzieh Salimi, Francesca Palombo, Pavel Matousek, Nicholas Stone

**Affiliations:** ^1^ School of Physics and Astronomy University of Exeter Exeter EX4 4QL UK; ^2^ Central Laser Facility STFC Rutherford Appleton Laboratory Oxford OX11 0QX UK

**Keywords:** diagnostics, gold nanostructures, light, photothermal therapy, theranostics

## Abstract

Nanotheranostics, which combines optical multiplexed disease detection with therapeutic monitoring in a single modality, has the potential to propel the field of nanomedicine toward genuine personalized medicine. Currently employed mainstream modalities using gold nanoparticles (AuNPs) in diagnosis and treatment are limited by a lack of specificity and potential issues associated with systemic toxicity. Light‐mediated nanotheranostics offers a relatively non‐invasive alternative for cancer diagnosis and treatment by using AuNPs of specific shapes and sizes that absorb near infrared (NIR) light, inducing plasmon resonance for enhanced tumor detection and generating localized heat for tumor ablation. Over the last decade, significant progress has been made in the field of nanotheranostics, however the main biological and translational barriers to nanotheranostics leading to a new paradigm in anti‐cancer nanomedicine stem from the molecular complexities of cancer and an incomplete mechanistic understanding of utilization of Au‐NPs in living systems. This work provides a comprehensive overview on the biological, physical and translational barriers facing the development of nanotheranostics. It will also summarise the recent advances in engineering specific AuNPs, their unique characteristics and, importantly, tunability to achieve the desired optical/photothermal properties.

## Graphical abstract

Nanotheranostics, which combines optical multiplexed disease detection with therapeutic monitoring in a single modality, has the potential to propel the field of nanomedicine toward genuine personalized medicine. A comprehensive overview of the recent advances in engineering gold nanoconstructs for nanotheranostics is provided. Their unique characteristics, required tunability to achieve the desired optical/photothermal properties and potential translational barriers faced are discussed.

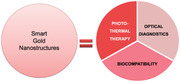

## 1 Introduction

Novel nanoparticle (NP) engineering has recently emerged as a key innovative driver in combining diagnosis and treatment into a “unified” one‐step platform known as “nanotheranostics.”^[^ ^1^, ^2^ ^]^ The application of this approach in cancer management is still in its infancy but promises to have significant potential, since state‐of‐the‐art optical technologies coupled with treatment modalities are expected to play an increasingly important role in disease management.^[^ ^3^ ^]^ However, more work is required to elucidate many of the mechanisms associated with its use in medicine.

An innovative formulation, such as a single agent or NP which can integrate the functions of imaging, targeting, and therapeutics in a single vehicle, is known as a theranostic agent.^[^ ^4^ ^]^ A theranostic agent has obvious clinical advantages over stand‐alone diagnostic or therapeutic entities; for example, a theranostic agent exploits specific biological pathways in the living system to detect and identify tumors and combine this with localized specific therapeutic action. Such agent could provide therapeutic delivery of light for elimination of heterogeneous tumor cells, by reducing the frequency of dosage and prolonging the therapeutic action of nanosystems in a safe, efficient, cost‐effective, and targeted manner.^[^ ^5^, ^6^, ^7^ ^]^ This specific information allows decisions to be made on the timing, quantity, type, and choice of treatment, as well as helping to assess and monitor a patient's response to treatment. The term “theranostics” was originally coined by John Funkhouser in 2002.^[^ ^8^, ^9^ ^]^ Theranostics representing the most important tools in diagnosis and treatment are becoming a rapidly growing and well‐established research field at the interface between nanotechnology and biomedical sciences. However, the first report of the use of theranostic platforms using radioactive iodine as the theranostic agent for imaging and treatment of thyroid cancer was published by Seidlin et al. in 1946,^[^ ^10^, ^11^ ^]^ and since then radioiodine therapy has become the gold standard in thyroid diseases.

The last decade has witnessed especially significant advances in the development of NPs for cancer diagnosis and treatment, with smart nanostructured materials having the potential to alter their structural, morphological, and functional features in response to specific internal (enhanced permeability and retention (EPR) effect, protein corona formation, passive targeting, exchange of ion channels) and external stimuli (electric or magnetic fields, electromagnetic radiation etc.).^[^ ^12^ ^]^ Initially, the use of these NPs was investigated for diagnosis and therapeutics separately. Despite their rapidly growing success in diagnosis and treatment, the safety and toxicity associated with such nanosystems have emerged as a serious concern and a potential barrier to the clinical translation of these nanoformulations.^[^ ^13^ ^]^ To address these safety challenges and to improve the targeted therapeutic efficacy, stable biocompatible nanosystems have also been explored.^[^ ^14^ ^]^ All these NPs and bioconjugated (functionalized) counterparts differ in their pharmacokinetic and toxicity profiles owing to their size‐dependent photoluminescent/plasmonic properties, shape, surface area, surface charge, aspect ratio, solubility, stability, structure and surface modification, and biodistribution in different organs.

In the last decade, there has been a significant advancement in developing NPs as two‐in‐one nanotheranostic agents.^[^ ^15^ ^]^ These may be classified in two types: the use and loading of extrinsically “switchable” optical agents onto the surface of NPs, known as “extrinsic nanotheranostic agents”; and the NPs with inherent features for both diagnosis and treatment, known as “intrinsic nanotheranostic agents.”^[^ ^16^ ^]^ Conventionally used NPs are extrinsic in nature, they include polymers, liposomes, and inorganic NPs and have limited penetration into tissues and low therapeutic efficacy.^[^ ^17^ ^]^ Conversely, intrinsic NPs, such as fluorescent quantum dots and plasmonic NPs are multifunctional, stable, simple, and straightforward. Noble metal NPs, such as gold NPs (AuNPs), have extensively been investigated as nanotheranostic agents, owing to their unique characteristics, versatility and tunable NIR surface plasmon resonance, excellent stability, low toxicity, high biocompatibility, and ease of surface conjugation.^[^ ^17^ ^]^

Detection and treatment of malignancies require a clear understanding of the disease and associated point of clinical need. Cancer progression and aggressiveness are typically classified into stages, where stage 0 means there is no cancer but only abnormal cells, stage I means the cancer tumor is small and only in one area, stage II and III mean the cancer tumor is larger and has grown into nearby tissues or lymph nodes, and stage IV means the cancer has spread to other parts of the body. For comprehensive classification, a system referred to as TNM (Tumor, Node, Metastasis) is used, where *T* refers to the size of the cancer and its spread into nearby tissue— it can be 1 (small), 2, 3, or 4 (large); *N* refers to whether the cancer has spread to the lymph nodes— it can be between 0 (no lymph nodes containing cancer cells) and 3 (lots of lymph nodes containing cancer cells); *M* refers to whether the cancer has spread to another part of the body—it can either be 0 (the cancer has not spread) or 1 (the cancer has spread).^[^ ^18^ ^]^ Therefore, typical detection procedures vary greatly depending on the type of cancer and may involve: physical examination of any tumor‐like abnormalities, laboratory tests for blood to detect unusual white blood cell count etc. Often these tests are insufficiently specific, thus leading to invasive collection of tissue samples (biopsies), which are then tested by histopathologists employing haemotoxylin and eosin (H&E) stain and microscopic images. They utilize the cellular features and tissue architecture to identify the presence of cancer and then provide a grade or stage of cancer. Cancer and precancer grading is subjective and prone to human error, in addition to the fact that such tests can require several days for the reporting process.^[^ ^19^, ^20^, ^21^ ^]^

There is a huge drive toward quantitative noninvasive instrument‐driven cancer detection imaging modalities; however, they are all still reliant on gold standard histopathology to provide the definitive diagnosis and grade of disease. Imaging tests like those based on computed tomography (CT), radio‐scintigraphy, magnetic resonance imaging (MRI), positron emission tomography, and X‐rays have gained importance in detection and staging of disease. All of these approaches have good depth of penetration for whole body imaging and a reasonable spatial resolution of the order of mm, however—with the exception of MRI—they all result in a potentially unnecessary radiation dose for the patient. On the other hand, optical imaging modalities, such as fluorescence, NIR, photoacoustic, optical coherence tomography (OCT) and Raman spectroscopy are nondestructive, use nonionizing radiation and therefore have no potential for inducing malignancies, can have high chemical specificity, but often a low penetration depth into tissue (mm to cm). This is because tissues are highly scattering as well as absorbing (at certain optical wavelengths) owing to components, such as water, lipids, melanin, and hemoglobin. The light absorption is considerably lower in the 650–950 nm spectral region, often referred to as the “optical window” or “tissue transparency window,” and this has paved the way to development of optical imaging modalities for cancer detection in this spectral range.

Surgery, radiotherapy, and chemotherapy are the current gold standard modalities to treat cancer, while photodynamic therapy (PDT), photothermal therapy (PTT), magnetic hyperthermia, immunotherapy, stem cell therapy, and combinations of these modalities are becoming accepted treatment methods for specific conditions and used as adjuncts to the gold standard methods.^[^ ^22^, ^23^, ^24^ ^]^ Although surgery is a very efficient and safe alternative, it has been unsuccessful in many cases,^[^ ^25^ ^]^ usually when a disease has advanced to a stage where the tumor has begun to grow outside of its primary site. On the other hand, chemotherapy is nonselective and nontargeted, meaning that the surrounding healthy tissue is also regularly damaged.^[^ ^26^ ^]^ The benefit of PTT compared to PDT and radiotherapy is that cell death is not dependent on the presence of oxygen, which may be depleted during treatment or may already be at low levels in hypoxic tumors.

Currently employed techniques for in vivo diagnosis are limited by a lack of specificity, and treatments are usually associated with systemic toxicity. Light‐triggered modalities could provide an appropriate nanotheranostic platform for multimodality tumor imaging in guiding the therapeutic process, such as PTT, which has recently gained increasing attention as an effective and safe approach.^[^ ^26^ ^]^ PTT offers a relatively noninvasive and gentle alternative for cancer treatment, using targeted AuNPs of specific shapes that absorb NIR light and produce localized heat for tumor ablation. These photoabsorbers (AuNPs) can be injected systemically or locally into the tumor to selectively increase the temperature under laser irradiation. Tumor destruction is thus achieved by raising the temperature to a sufficient level over a required period. In this therapeutic strategy, the photon energy is converted into heat and, once the temperature exceeds ≈42 °C for a sufficiently long time, this will induce localized cell death. A commonly accepted “rule of thumb” when considering heating for hyperthermia relates to the thermal dose unit. This is defined based on the cumulative equivalent minutes of exposure at 43 °C causing approximately half of cell to die, when cells are exposed to this temperature for 60 min.^[^ ^27^, ^28^ ^]^ Thermal dose units have been derived from the observation, in many cell types, that above 43 °C a similar level of cell damage is achieved in approximately half the time when the temperature is increased by 1 °C. PTT does not have the same potential for severe infection that can be encountered after surgery. It also overcomes the side effects of chemotherapy by circumventing the use of systemically toxic drugs.^[^ ^29^ ^]^ Furthermore, unlike in radiotherapy and PDT, the presence of oxygen is unnecessary in PTT to induce cell death.

Currently, many photothermal agents, such as noble metal nanostructures, transition metal chalcogenides and oxides, carbon‐based materials, and organic compounds have been widely investigated.^[^ ^30^ ^]^ Furthermore, AuNPs have great photothermal theranostic effects, strong localized surface plasmon resonance and photostability in contrast to other agents.^[^ ^31^ ^]^ Gold nanostructures have received a great deal of attention as theranostic agents due to spatiotemporal selectivity and specificity for disease destruction when functionalized with targeting moieties, ease of surface functionalization/modification, low toxicity, high biocompatibility, high surface‐to‐volume ratio, optical properties, and interplay of size‐ and shape‐dependent NIR imaging and therapeutic efficacies.

AuNPs have been produced in different shapes, such as nanoshells,^[^ ^16^ ^]^ nanorods (NRs)^[^ ^32^ ^]^ nanocages,^[^ ^33^ ^]^ nanostars,^[^ ^17^ ^]^ nanospheres,^[^ ^32^ ^]^ and core–shell structures.^[^ ^34^ ^]^ Localized surface plasmon resonance (LSPR) wavelengths of AuNPs can be tuned from the visible to the NIR region by changing the size and shape of NPs. AuNPs can be functionalized to make them selective and targeted toward diseased tissues, and have been explored as multiplexed contrast agents for several diagnostic techniques (such as computed tomography,^[^ ^8^ ^]^ surface enhanced Raman scattering (SERS),^[^ ^7^ ^]^ and photoacoustics^[^ ^6^ ^]^) coupled with PTT for the diagnosis and treatment of cancer.^[^ ^22^, ^23^, ^24^, ^25^, ^26^ ^]^ Each of these diagnostic techniques has its own merits and limitations in visualizing a tumor for targeted PTT based on their specificity, sensitivity, potential to detect and provide image‐guided therapeutic processes, and in situ excitation of theranostic agents.^[^ ^35^ ^]^

Apart from AuNPs, there are various different classes of photothermal theranostic agents including transition metal chalcogenides and oxides (e.g., CuxSy, Cu_2_–xSe, MoS_2_, WS_2_, FeSe_2_, FeS, TiS_2_, MoOx, WxOy, Ti_8_O_15_ with different structures, e.g., nanosheets, nanodots, and spherical NPs), carbon‐based materials (such as single‐walled carbon nanotubes, pristine graphene, graphene oxide, reduced graphene oxide, functionalized graphene quantum dots, graphene nanoribbons), organic materials as well as organic dye loaded micelles, liposomes, and protein‐based nanocomposites (polymers of polyaniline (PANI), polypyrrole (PPy), and poly(3,4‐ethylenedioxythiophene):poly(4‐styrenesulfonate) (PEDOT:PSS)).^[^ ^36^, ^37^ ^]^ A review of the development of these photothermal theranostic agents is beyond the scope of this discussion but has been reported elsewhere.^[^ ^38^ ^]^ The diagnostic ability and photothermal heating efficiency of transition metal chalcogenides and oxides are reduced when their size is decreased, which limit their further biomedical application.^[^ ^39^ ^]^ In the case of carbon‐based materials, the poor dispersibility and stability in biological solutions have been shown to induce side effects and enhanced toxicity profile, which may restrict their further application.^[^ ^40^ ^]^ However, this challenge can potentially be addressed by modifying the surface of graphene with polyethylene glycol (PEG), polyacrylic acid, and other hydrophilic groups.^[^ ^41^ ^]^ Organic materials and dyes have been shown as promising photothermal theranostic agents, due to the high absorption cross‐section and fluorescent emission that can be utilized for tumor detection and high NIR photothermal effects. However, long‐term toxicity and photobleaching and unclear biodegradation mechanisms limit their further application in nanotheranostics.

Due to the tunable and controllable LSPR of AuNPs, providing a number of functions, such as photothermal conversion and SERS activity, they make an ideal plasmonic material for use in the development of nanotheranostics for personalized medicine. The LSPR can be modified adjusting the aspect ratio, size, shape, and aggregation. These unique plasmonic characteristics of AuNPs can be employed in PTT by the transfer of electrons from the conduction band of NPs and their fast deactivation through e–e scattering, to produce high‐potential‐energy “hot” electrons which in turn induce localized heat in tissues to initiate tissue ablation.^[^ ^42^, ^43^ ^]^ Recently, efforts to raise SERS signals and the conversion of photon energy to thermal energy have mainly focused on size, shape, surface chemistry, and LSPR tunability of AuNPs.^[^ ^44^ ^]^ Gold nanostructures have recently been exploited as multifunctional nanotheranostic systems for simultaneously obtaining cell‐targeted SERS imaging and PTT. In vitro and in vivo findings suggest that smart and versatile gold nanostructures are promising NIR light‐triggered and targeted theranostic platforms for imaging‐guided PTT of cancer, which may provide a solution to the bottleneck problems of both diagnosis and treatment, including limited penetration depth and oxygen‐deficient microenvironments.^[^ ^45^, ^46^, ^47^, ^48^, ^49^, ^50^ ^]^

In this review, we will survey recent advances in plasmon‐assisted gold nanostructures. Section 2 will give the readers the basics of nanoplasmonics, synthesis, morphology, and unique features of gold nanostructures. We will successively describe the optical and thermal properties of AuNPs. In Section 3, we will highlight the potential dark‐ and phototoxicity, biological fate, biodistribution, and cellular uptake of these nanostructures to target tumors while remaining nontoxic to normal cells. Sections 2.5 and 2.6 will provide a snapshot on the application of AuNPs in a wide spectrum of diagnosis (fluorescent, magnetic resonance, photoacoustic imaging, Raman spectroscopy) and treatment (PTT), with a special focus on thermal biology of AuNPs. In Sections 4, we will review the in vitro and in vivo studies in which photothermal theranostic approaches have been reported. Finally, Section 5 will highlight the barriers and challenges in translating the AuNPs into clinical settings, with a focus on future perspectives for triggering chemical transformation of AuNPs to enhance the efficiency of light‐triggered nanotheranostic modalities.

## 2 Designing Smart Gold Nanostructures

**Figure 1:**
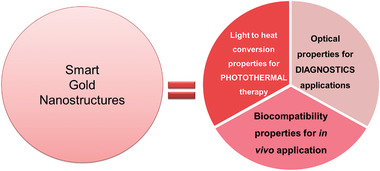
Schematic diagram showing the important functionalities of gold nanostructures.

Cancer theranostics has been a key research area in the past decade and is growing in importance, as researchers around the world are now able to more effectively bridge diagnostic and therapeutic strategies. Though the term “theranostics” has already been in use for some time now, it has proved to be challenging to develop a single platform providing the best of both worlds. This has given rise to multiple components combined into one nanostructure in complicated strategies, unleashing various combinations of diagnostic and therapeutic approaches, hence diversifying as well as defocussing the outcome. To this end, gold nanostructures have been one of the key single‐mode platforms, realizing the concept of theranostics with diagnostics supported by its optical properties, therapy provided by the photothermal properties and its inherent biocompatibility (compared to quantum dots, iron oxide NPs etc.) for use in vivo. This concept has schematically been depicted in **Figure** **1**. Many research groups worldwide are working on the design of gold nanostructures for smart theranostics. As these need to be employed in vivo, secondary functionalities are needed in order to provide active targeting to specific sites and/or passive targeting due to the EPR effect, higher blood circulation times due to good biocompatibility, etc. Primarily, after administration (oral, through injection at the site or intravenous), the nanostructures are tracked or imaged by a diagnostic modality (namely, noninvasive optical spectroscopies) to detect the specific subcategory of disease depending on the employed functionalization (molecular targeting groups on the nanostructures). A potential way to detect and identify heterogeneous diseases simultaneously, as well as the stage of the disease, would be to employ multiplexed (multiple targeting group‐diagnostic label pairs) gold nanostructures. Following this, should a specifically defined signal be obtained, the therapy would be triggered specifically at the disease site. The diagnostic modality should thereafter also allow monitoring of the effectiveness of the therapy. Achieving this goal would help in providing personalized treatments to patients.

### 2.1 Why Gold Serves as an Efficient Photothermal Agent?

PTT is referred to as a process which involves the selective heating of the local environment in which the PTT agent is employed. In particular, common PTT agents absorb light hence promoting electronic transitions from ground to excited states, followed by a nonradiative decay leading to heating of the local environment of the PTT agents. PTT agents, such as natural chromophores and light‐absorbing dyes have relatively low absorption cross‐section, poor light‐to‐heat conversion, and photobleaching, thereby making them inefficient as potential clinical theranostic agents. In contrast, plasmonic nanostructures benefit from high light‐to‐heat conversion and eliminate the possibility of photobleaching. These structures have readily induced surface plasmons (oscillations of the conduction band electrons at the nanoparticle surface) that can be resonant with the incident light thus producing an LSPR. Heat is produced by resistive heating from the oscillating electrons within the surface of the metal, when light energy is absorbed by the NP, which typically depends on the LSPR profile of the NP.

It is important to consider the temperature rise required for triggering cell death and hence for application in cancer therapy. It has been suggested that cell death can be induced by increasing the body temperature to a hyperthermic temperature of 42–47 °C.^[^ ^51^, ^52^ ^]^ It is worth noting that the induced temperature rise, even when it is due to similar nanostructures, may vary depending on their difference in heat absorbing and dissipating environments (aqueous environment, in vitro or in vivo), heat transduction and local thermal conduction properties, thereby making it difficult to compare directly their efficiencies in different environments.

Hence, in order to utilize gold nanostructures effectively in PTT, it is vital to be able to specifically tailor them for use. PTT would require gold nanostructures to primarily provide an efficient light‐to‐heat conversion by first maximizing the light absorbed by the nanostructures while avoiding significant loss of energy due to phenomena, such as scattering, fluorescence, phosphorescence, etc.^[^ ^23^, ^32^, ^36^ ^]^ In this perspective, it is vital to maximize the light absorption of the metal nanostructures at and around the wavelength that can be medically used to trigger therapy in patients, which in turn depends on the tissue transparency window 650–950 nm, i.e., a region of highest penetration through the tissue.^[^ ^53^, ^54^ ^]^ Hence, gold nanostructures have been prepared in such a way that the LSPR overlaps with the tissue transparency window. Another important consideration is the laser excitation line to be used for this purpose. Most studies report the use of 808 nm excitation line and hence gold nanostructures with LSPR peak positions in vicinity of the laser line would provide an enhancement in the performance of the nanostructures (detailed discussion and examples in Section 2.3). It should be noted that the optical extinction of gold nanostructures stems from scattering and absorption, where absorption dominates in most cases. Detailed understanding of the relationship between these parameters can be found in the reports by Jain^[^ ^53^ ^]^ and Hu.^[^ ^54^ ^]^

**Figure 2:**
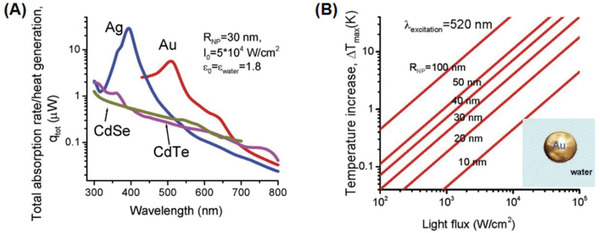
Photothermal properties of NPs based on A) material (other parameters identical) and B) size. Adapted with permission.^[^ ^55^ ^]^ Copyright 2007, Elsevier.

Govorov and Richardson^[^ ^55^ ^]^ have discussed in great detail the correlation between nanostructure design (for simplicity, its radius) and temperature increase (Δ*T*). The equation below provides the temperature increase as a function of distance, *r*, from the center of a single NP

(1) $$\Delta T\left(r\right)=\frac{V_{\mathrm{NP}}Q}{4\pi k_{0}r}$$

where *V*_NP_ is the NP volume, and *Q* or *Q*(*r*_np_,*t*) is a function of the NP radius, *r*_np_, and time, *t*, and *k*_0_ is the thermal conductivity of the surrounding medium. This expression is valid outside the NP, i.e., for *r* > *r*_np_, and is the calculated heat generation (assuming that the incident light wavelength is much longer than the NP radius). The maximum temperature rise occurs at *r* = r_np_ and hence the maximum temperature can simply^[^ ^55^ ^]^ be given as ∆*T*_max_ *α r*_np_^2^. **Figure** **2**A features the total heat generation (*q*_tot_ = *V*_NP_ *Q*) for both plasmonic and semiconductor NPs. Typical semiconductors (e.g., CdSe and CdTe) exhibit low heat generation rates compared to those of plasmonic NPs, justifying the use of plasmonic NPs as PTT agents. For plasmonic NPs, the heat generation rate mimics their plasmon behavior.^[^ ^55^ ^]^ Additionally, silver presents a tenfold increased heat generation compared to gold at their respective plasmon peaks and for identical parameters, i.e., *r*_np_ = 30 nm, light flux *I*_0_ of 5 × 10^4^ W cm^−2^ and surrounding medium, as shown in Figure 2A.^[^ ^55^ ^]^ This heat generation slowly tails off in the NIR window where gold shows a slightly better performance than silver, giving gold an edge especially for in vivo theranostic applications. Furthermore, the instability of silver (especially in biological media) also discourages its use, establishing gold as a superior choice as PTT agent. Figure 2B shows a temperature rise with increasing a) light flux and b) NP size, when the wavelength of light is tuned to match the LSPR peak maximum. This is also supported by the experimental reports by Qin et al.^[^ ^56^ ^]^ which show up to two orders of increase in absorption cross‐section, *C*_abs_, with increasing NP size from 15 to 100 nm, thereby confirming the significance of size in heating efficiency.

### 2.2 Synthesis of Gold Nanostructures for Theranostics

Although both top‐down and bottom‐up approaches have widely been used for nanostructure design, the bottom‐up approach of forming gold nanostructures from gold atoms (gold salt reduction methods) has been popular for preparing colloidally‐stable structures required for in vivo applications. There have been numerous reports and reviews^[^ ^57^, ^58^, ^59^, ^60^, ^61^, ^62^ ^]^ focusing on gold nanostructure synthesis to which the reader is directed. **Table** **1** lists the variety of gold nanostructures that have been reported in the literature and most importantly reflects on their tunability of shape, size, etc., to manipulate the optical properties, especially LSPR and photothermal properties (ratio of absorption to scattering coefficient *μ*_a_/*μ*_s_).

**Table 1:** Nanostructures and their tunable properties

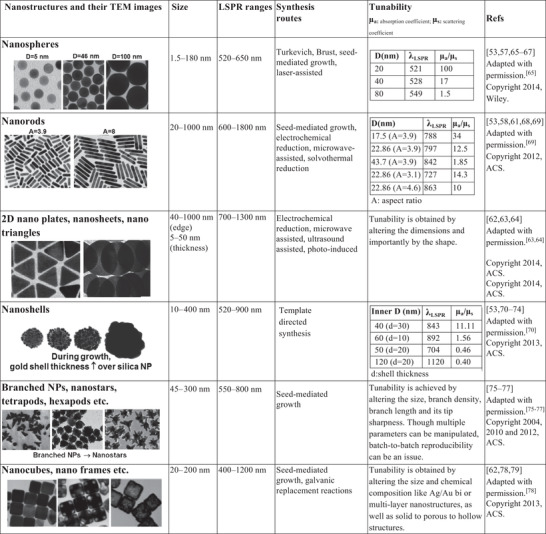

**Figure 3:**
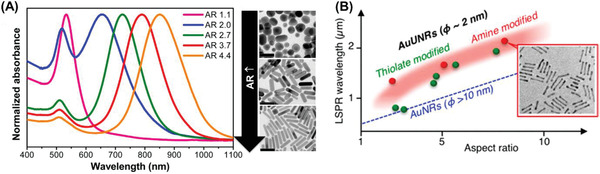
A) Nanorod aspect ratio‐dependent LSPR peak in the near‐IR region. Adapted with permission.^[^ ^82^ ^]^ Copyright 2014, American Chemical Society. B) LSPR in mid‐IR with ultrathin NRs. Adapted with permission.^[^ ^80^ ^]^ Copyright 2018, American Chemical Society.

Nanorods, nanoshells, branched nanostructures, and nanoassemblies have gained popularity in this field and demand a more detailed overview. In contrast to spherical NPs, NRs inherently feature a longitudinal LSPR in the NIR region and can be tuned by manipulating their size and aspect ratio (AR), as shown in **Figure** **3**. Recently, Takahata et al. have reported ultrathin NRs with an AR of about 10–20 that exhibit LSPR in the mid‐IR (MIR) region (see Figure 3B).^[^ ^80^ ^]^ Although their LSPR overlaps with the transparency window which should translate into higher photothermal efficiency, the typical polydispersity of the NRs (in a synthesized batch) is higher than that of the spherical NPs, effectively lowering their average absorption cross‐section.^[^ ^56^ ^]^ Another criterion for consideration is that heating causes melting and reshaping of the NRs, yielding NRs with lower AR and LSPR than the original, potentially making them impractical for therapeutic use as they would change their light‐heat transduction efficiency during a treatment cycle. Murphy and co‐workers^[^ ^68^, ^81^, ^82^ ^]^ have elaborately investigated and reported this effect.

**Figure 4:**
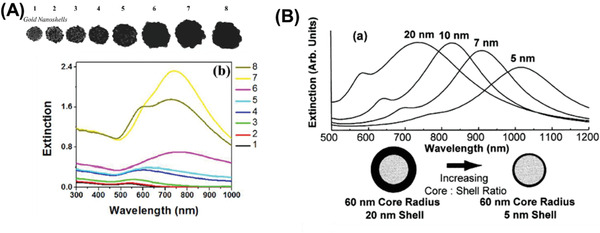
Manipulating nanoshell properties. A) Evolution of nanoshell growth on a silica nanoparticle (TEM and UV–vis). Adapted with permission.^[^ ^70^ ^]^ Copyright 2013, ACS. B) Theoretically calculated optical resonances of metal nanoshells silica core, gold shell over a range of core radius:shell thickness ratios. Adapted with permission.^[^ ^73^ ^]^ Copyright 1998, Elsevier.

Other nanostructures that have become popular are gold nanoshells and gold nanostars as their LSPR peak can be positioned in the NIR region. Manipulating the shell thickness in the range of 1–30 nm for gold nanoshells and the number of branch, tip sharpness, length of the tip for gold nanostars provides a handle over its *λ*_LSPR_ tunability. Nanoshells^[^ ^70^, ^73^ ^]^ were synthesized initially onto dielectric silica cores, and the stagewise growth of the shell involved formation of the gold seeds which later coalesced into the silica sphere (having nanoroughness) finally giving rise to a continuous gold shell over the silica core (as depicted in **Figure** **4** and **Table** **1**). With an increase in shell thickness, the LSPR is blueshifted. Thus, a shell thickness of 2–10 nm was ideal to provide a NIR LSPR peak, as depicted in Figure 4B.^[^ ^73^ ^]^ However, upon completion of the shell formation, a secondary peak at a lower wavelength is observed, in addition to the primary LSPR peak, with reduced extinction as compared to its previous partial shell stages.^[^ ^70^ ^]^ Recently, various nanomaterials, such as Pt/Pd^[^ ^72^ ^]^ or magnetic NPs^[^ ^83^ ^]^ have been utilized as cores to provide added functionality to the hybrid nanostructure system.

**Figure 5:**
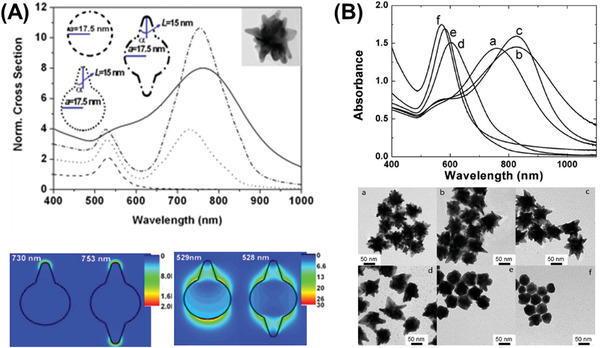
LSPR dependency on nanostar morphology. A) Star tip‐dependent LSPR. Adapted with permission.^[^ ^83^ ^]^ Copyright 2011, Wiley. B) Synthesis conditions manipulated for change in tip sharpness and thereby LSPR. Adapted with permission.^[^ ^85^, ^86^ ^]^ Copyright 2010, American Chemical Society; and Copyright 2012, IOP.

Interesting nanostructures, such as branched gold NPs and nanostars have also gained popularity. Marzán and co‐workers have strategically employed poly(vinylpyrrolidone) (PVP) and dimethylformamide (DMF) to obtain gold nanostars^[^ ^84^, ^85^ ^]^ with tunable dimensions and optical properties. By varying reactant ratios and synthesis temperatures, they have reported a wide range of minimally branched morphologies to highly spiked nanostar morphologies. **Figure** **5** illustrates the ability to manipulate the LSPR peak position by controlling the different aspects of the nanostar morphology, which can be obtained by varying and optimizing the synthesis temperatures, reactants, reactant ratios, etc. Studies suggest NIR LSPR peaks were obtained for nanostars of 100–200 nm and more with high polydispersity. Though such nanostructures have shown promising results, such size ranges hinder their applications in vivo due to their low blood circulation times resulting lower effectiveness in theranostic applications. Although there are controversies on the exact size values, Blanco and co‐workers^[^ ^12b^ ^]^ suggest that spherical gold NPs>150 nm in diameter, nanoplates, and NRs and positively charged NPs have a higher tendency to accumulate in the lungs, liver, and spleen, whereas, spherical 5 nm NPs accumulate more readily in the kidneys, i.e., have higher chances of being excreted from the body. This is discussed further in Section 3.

**Figure 6:**
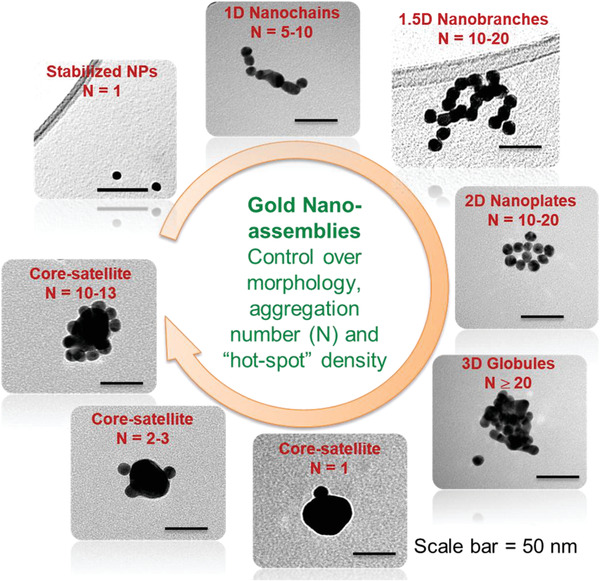
Designing and controlling gold nanoassemblies. Adapted with permission.^[^ ^87^, ^91^, ^92^ ^]^ Copyright 2013 and 2014, ACS; and Copyright 2014, RSC.

Additionally, arranging NPs into nanoassemblies^[^ ^86^, ^87^, ^88^, ^89^, ^90^, ^91^, ^92^ ^]^ also suffices to redshift the *λ*_LSPR_ into the near‐NIR 600–800 nm region. As the nanoassemblies should also satisfy the requirement of being sub‐100 nm in size, hence dimers of 40 nm, NP assemblies of smaller sizes (about 15 nm diameter) and combinations thereof have become relevant. These customized assemblies contain multiple nanojunction “hot‐spots” featuring high electric fields and redshifted LSPR. Some of such morphologies have been shown in **Figure** **6** which exhibit LSPR of about 615–750 nm.

Core‐satellite morphologies allow controlling the satellite density and hence hot‐spot density, along with the use of any shaped core (spherical, NRs, nanostars, etc.) or satellite (spherical or NRs).^[^ ^91^, ^93^, ^94^ ^]^ Nanobranched morphologies have gained popularity due to their relatively broad NIR absorbance.^[^ ^88^ ^]^ Such nanoassemblies offer the added advantage of boosting the temperature increment of the surroundings over and above that of the individual NPs.^[^ ^95^ ^]^ It is worth mentioning that, with more complicated nanostructure shapes and designs, both polydispersity (e.g., as reported for branched nanoantennas^[^ ^96^ ^]^) and synthesis scale‐up becomes an issue.

### 2.3 Manipulating Photothermal Efficiency of Gold Nanostructures

**Figure 7:**
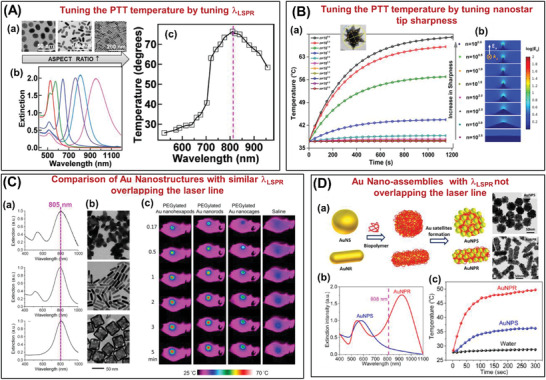
A) Example of tuning the PTT temperature by tuning LSPR peak position. a)TEM images of gold NRs with different aspect ratios, b) their respective UV–vis spectra, c) temperature increase as a function of LSPR peak positions with 809 nm laser line (marked in dotted line). Adapted with permission.^[^ ^97^ ^]^ Copyright 2013, American Chemical Society. B) Example of tuning the PTT temperature by tuning the tip sharpness of gold nanostars. a) Temperature‐time plot of nanostars with increasing tip sharpness along with TEM image, b) Electric field intensity plot with increase in tip sharpness of the gold nanostars. Adapted with permission.^[^ ^98^ ^]^ Copyright 2015, American Chemical Society. C) Comparison of different morphological gold nanostructures with similar LSPR peak positions. a,b) UV–vis spectra and TEM images of gold nanohexapods, NRs, and nanocages, c) thermographs of tumor‐bearing mice receiving photothermal treatment for different periods of time. The mice were intravenously administrated with aqueous suspensions of PEGylated nanohexapods, NRs, nanocages, or saline. Adapted with permission.^[^ ^99^ ^]^ Copyright 2018, American Chemical Society. D) Example of core‐satellite gold nanoassemblies with off‐resonant LSPR peak positions. a) Schematic representation of the formation of such nanoassemblies with a nanosphere as the core (AuNPS) and nanorod as the core (AuNPR) along with the TEM images of the prepared nanoassembly structures, b,c) their respective UV–vis and temperature‐time plots of the respective nanoassembly structures. Adapted with permission.^[^ ^100^ ^]^ Copyright 2010, Wiley.

This section discusses the effect of the nanostructure design in maximizing the photothermal temperature increase with some relevant examples. Particularly, we discuss certain nanostructures that have gained prominence for use in PTT like NRs and nanostars along with their structure‐photothermal relation. Chen and co‐workers^[^ ^97^ ^]^ report interesting correlations between gold nanorod structures, their optical and photothermal efficacy by undertaking a series of experiments, where temperatures have been recorded from NP colloids with a thermocouple after exposure to laser. They demonstrate LSPR dependency of the photothermal properties of gold nanostructures with 809 nm laser excitation. **Figure** **7**A shows that among a variety of gold NRs with LSPR ranging 600–950 nm (a and b), the highest temperatures were achieved for gold NRs with an LSPR coinciding with the laser line (marked with dotted line in Figure 7Ac)). They also suggest that coating gold nanostructures with strongly light absorbing materials like (Ag_2_S, ZnS, etc.) result in LSPR shift to NIR region and help in improving the photothermal conversion efficiencies. It is important to point out that though Ag_2_S, ZnS, etc., aid in light absorption, it negatively impacts the potential for diagnostic applications using surface enhanced Raman signals and therefore may not be an ideal solution.

Furthermore, NP volume, assembly nanostructures, shell coating have also been reported as factors influencing photothermal properties. Among others, nanostar morphologies have been investigated in great detail. Chatterjee and co‐workers^[^ ^98^ ^]^ studied nanostars and emphasize the importance of tip sharpness in escalating the surrounding temperature (as depicted in Figure 7B)), thereby promoting higher cell death. Laser heating of the nanostructures incubated at the tumor site was carried out with a continuous wave infrared diode laser (5.0 mW) at 785 ± 5 nm for 20 min. Temperature rises of ≈30 °C has been reported at the sharpest tips within 20 min time. In contrast, lowering of tip sharpness resulted in redshifted LSPR peak, beyond 785 nm, which dramatically hampered its photothermal performance, i.e., the temperature increase of less than a few degrees observed. Importantly, a study by Wang and co‐workers^[^ ^99^ ^]^ explicitly compares different morphologies of gold nanostructure and their PTT temperature increase behavior. Figure 7C) features gold nanohexapods, NRs, and nanocages with almost identical LSPR peak position (coinciding with the laser line of 805 nm, pink dotted line in figure) and demonstrates that although all the nanostructures aid in temperature rise, the nanohexapods perform better when used in a mouse tissue model. Particularly, the NRs are reported to reach 4 °C higher than the nanocages, while the nanohexapods attain 2–3 °C higher than the NRs, at 5 min of exposure each.

Researchers have also been interested in nanoassemblies as alternative nanostructures.^[^ ^45^, ^48^ ^]^ An interesting study by Tian and co‐workers^[^ ^100^ ^]^ compare the core‐satellite nanoassemblies with spherical gold cores (AuNPS), as well as gold nanorod cores (AuNPR), as shown in Figure 7D(a). The off‐resonant nanoassembly structures (LSPR peak position does not coincide with the laser excitation of 808 nm) provided temperature increase of AuNPR solution (Δ*T* = 24 °C) compared to AuNPS solution (Δ*T* = 8 °C) and water (Δ*T* = < 1 °C). This can be attributed to the around three times higher absorbance at 808 nm for AuNPR than AuNPS (UV–vis spectrum b and temperature plot c). Tuning the absorbance of gold nanostructures to the NIR II band (1000–1350 nm) for PTT have been attempted by creating black body type nanostructures with significant absorption in both NIR I and NIR II.^[^ ^100^ ^]^ It can thus be fairly concluded that the highest photothermal efficiency can be obtained with nanostructures featuring the highest absorbance at the laser excitation wavelength. It is important to realize that for a true comparison of efficiency, the temperature rise is dependent on a multitude of parameters that can affect the absolute increase in temperature.

### 2.4 Thermal Dosimetry

There is a commonly accepted “rule of thumb” when considering heating for hyperthermia which relates to the thermal dose unit. This is based on the cumulative equivalent minutes of exposure at 43 °C, whereby, when cells are exposed to this temperature for 60 min, approximately half of cell will survive.^[^ ^26^, ^27^ ^]^ Note, this is based entirely on the cell type and needs to be ascertained for the cell type targeted. However, thermal dose units have been derived from the observation, in many cell types, that above 43 °C a similar level of cell damage is achieved in approximately half the time, when the temperature is increased by 1 °C. Sapareto and Dewey^[^ ^101^ ^]^ suggested the use of “degree‐minutes” as the dose calculator which converts all thermal exposures to “equivalent‐minutes.” They developed a formula where the time at 43 °C, *t*_43_ = t*R ^(43‐T)^, where, *t* is the time in minutes at *T* °C and *R* = 0.5 for *T* > 43 °C and *R* = 0.25 for *T* < 43 °C.

### 2.5 Gold Nanostructures as Optical Diagnostic Agents

With the focus on “theranostics” and an aim in making clinical care both more effective and reduce procedures for patients to undergo, as compared to employing two separate agents for diagnosis and therapy, gold nanostructure‐based PTT needs to complement the associated diagnostic modality. Below we will discuss briefly the different diagnostic modalities supported by gold nanostructures and provide some examples thereof. Gold nanostructures being plasmonic can be employed for optical‐based diagnostic modalities like fluorescence, Raman, photoacoustic imaging, etc., in addition to more commonplace techniques such as X‐ray CT.

#### 2.5.1 X‐Ray CT Imaging

**Figure 8:**
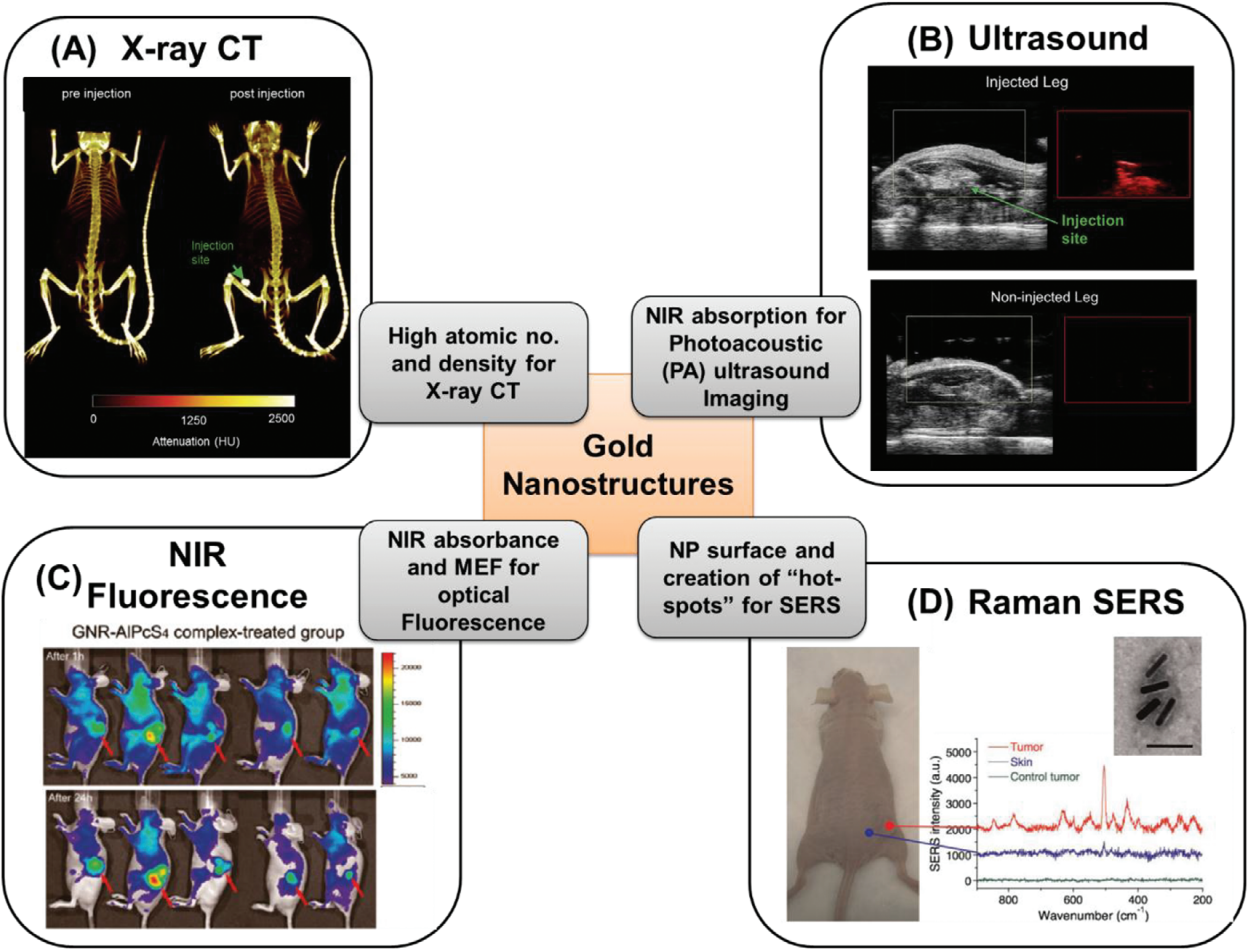
Examples of gold nanostructure employed in optical diagnosis in mice tumor models by injecting the customized gold nanostructures, along with featuring their specific property for specific diagnosis. A) X‐ray CT imaging demonstrating tumor detection (marked with arrow) for pre‐ and postinjection. Adapted with permission.^[^ ^106^ ^]^ Copyright 2016, Elsevier. B) Ultrasound imaging demonstrating tumor detection (marked with arrow) in injected and noninjected leg of mice. Adapted with permission.^[^ ^106^ ^]^ C) NIR fluorescence imaging with coated gold NRs after 1 and 24 h demonstrating tumor detection (marked with arrow). Adapted with permission.^[^ ^107^ ^]^ Copyright 2011, American Chemical Society. D) Raman (SERS‐based) spectroscopy employing gold NRs demonstrating tumor detection by comparing the spectrum of skin, tumor, and control tumor. Adapted with permission.^[^ ^108^ ^]^ Copyright 2010, Wiley.

X‐ray CT imaging is an important form of noninvasive 3D imaging due to its almost universal availability in medical clinics and hospitals, making infrastructure implementation and acceptability easy. Contrast in X‐ray imaging is derived from the difference in mass attenuation between two tissues. Materials with a high atomic number or density, such as bone absorb more X‐rays, making them detectable. Contrast agents play an important role in allowing higher energy, safer scans with high contrast by introducing high atomic number media like iodine or gold colloid into the body. The attenuation is often measured in Hounsfield units (HU) which is normalized to the attenuation of water (= 0 HU) and scales linearly.^[^ ^102^ ^]^ The HU values between different research groups are not directly comparable as it heavily depends on the energy of the X‐ray scan. Iodinated contrast medium (concentrations of 150–400 mg mL^−1^ iodine) has been employed as the primary X‐ray contrast agent till date. Research has provided good knowledge in the various aspects where gold nanostructures^[^ ^102^, ^103^ ^]^ could be utilized. Maltzahn and co‐workers^[^ ^104^ ^]^ report the benefits of PEG—protected gold NRs as X‐ray contrast agents. Gold NRs with superior spectral bandwidth and optical conversion efficiency for photothermal heat generation provides an improved circulation half‐life in vivo (*t*_1/2_ ≈17 h, compared with gold nanoparticles (NPs) and gold nanoshells) and importantly ≈ two fold higher X‐ray absorption than clinical iodine contrast agent. On the other hand, Ma et al.^[^ ^105^ ^]^ report shape‐independent X‐ray absorption for gold nanospheres, nanospikes, and NRs per mass of Au cellular uptake, but points out that the cellular uptake and hence the X‐ray efficiency trend followed nanospheres > nanospikes > NRs. Typically, gold concentrations of 100 mg Au mL^−1^ has been studied. **Figure** **8**A demonstrates the X‐ray contrast obtained with gold nanostructures and quantifies them in HU units.

#### 2.5.2 Photoacoustic (PA) and Associated Ultrasound Imaging

PA imaging uses pulsed light to transiently heat a local absorber, which could be red blood cells or exogenous (NPs) causing heating of the local surrounding, which upon thermal expansion of the tissue causes a pressure wave that is detected through ultrasound. Laser sources typically used in photoacoustic imaging comprise of Q‐switched, pulsed Nd:YAG lasers providing several tens or hundreds of mJ at 1064, 532, or 355 nm, pumping broadband optical parametric oscillators to give tunable nanosecond pulses across the visible and near infrared spectral region. Ultrasound imaging, on the other hand, is based around the creation of acoustic waves in the tissues and measuring how they are reflected and attenuated by the tissues and their interfaces.

Employing light in the NIR window for PA is advantageous and as such gold nanostructures featuring NIR absorbance are ideal candidates. Therefore, gold nanostructures designed by varying size or morphology (e.g., nanospheres, nanoshells, nanocages, and NRs) have been utilized by this imaging technique in the research phase.^[^ ^109^ ^]^ As tumors are often quite small, PA and ultrasound techniques do not always provide sufficient spatial resolution or specificity required for a full diagnosis. However they are often able to identify abnormalities for further investigation and are relatively cheap, as compared to whole body scanners, such as MRI and X‐ray CT and hence have an advantage. Typically, the NP coating plays an important effect on imaging efficiency, for example, in perfluorooctyl bromide (PFOB) coated gold nanoshells,^[^ ^110^ ^]^ PFOB with higher acoustic impedance than air, thus boosting ultrasound imaging. An example is shown in Figure 8B. On the other hand, gold nanobranched structures, gold nanostars, gold NRs have also been exploited for PA imaging.^[^ ^111^ ^]^ Targeted gold (NPs) in the concentration of 10^8^–10^9^ NPs mL^−1^ exhibited significant PA signals when compared with nontargeted AuNPs and the ADS740WS NIR dye in cell/gelatin samples at 680 nm wavelength illumination.^[^ ^112^ ^]^ Recently, a switchable PA imaging agent has been reported by Kim et al.^[^ ^113^ ^]^ with gold NRs and the presence or absence of the silver coating onto the gold NRs provided a PA signal off or on, respectively. This was demonstrated in vivo by IV injection of silver etchant into the previously injected silver‐coated gold NRs, where the PA signal would switch on after reaction with the etchant.

#### 2.5.3 NIR Fluorescence Imaging

Fluorescence dyes and more recently quantum dots have become popular as fluorescent agents. First reported by Mooradian,^[^ ^114^ ^]^ the fluorescence of bulk gold, was observed to be very weak with a quantum yield of the order of 10^−10^ and hence was limited in its application. But lately, strong fluorescence with a quantum yield of up to 10^−3^ has been reported for gold (NPs) and nanoshells, paving way for their application as NIR fluorescence agents.^[^ ^115^, ^116^, ^117^ ^]^ Additionally, gold nanostructures feature benefits of no photobleaching, observed with other dyes, and depending on the phenomenon exploited gold can even enhance fluorescence of certain organic dyes utilizing the phenomenon of metal‐enhanced fluorescence. Figure 8C depicts a study where tailored gold nanostructures have been employed to detect tumor (marked with arrow) where the higher NP uptake into the tumor can be readily observed.

#### 2.5.4 Raman Spectroscopy

Last but potentially most importantly, there has been tremendous work in the field of employing gold nanostructures for SERS diagnosis. Conventional (normal) Raman signals are molecular specific and able to distinguish pathological tissues and cells,^[^ ^118^, ^119^, ^120^ ^]^ however, they are inherently weak and therefore without the use of fiber optics to deliver laser light and efficiently collect Raman signals,^[^ ^121^, ^122^ ^]^ it is unlikely that Raman will be able to probe diseases in deep solid organs, although deep Raman methods are showing some promise.^[^ ^123^, ^124^ ^]^ However, the SERS phenomenon of boosting Raman signals of a molecule (acting as a label or tag) when positioned at a “hot‐spot” (NP surface or NP–NP junction) can be well‐utilized as an optical diagnostic technique. It utilizes the enhancement of electric field around plasmonic nanosurfaces, which can be further amplified at a NP–NP junction (in plasmonic nanoassemblies) and are referred to as hot‐spots.^[^ ^91^, ^92^ ^]^ When a molecule with high Raman cross‐section (often small aromatic molecules) sits in such a hot‐spot, the inherent Raman signals of the molecule are dramatically enhanced up to 4–8 orders of magnitude in signal intensity. The noble metal plasmonic substrate, most often Au or Ag, acts as the SERS signal amplifier. This SERS enhancement of molecules has been either applied in chemo/biosensing, i.e., unknown molecule brought close to SERS amplifier for detection and/or quantification, or known molecules labeled onto SERS amplifiers and its location tracked for detection of tumors.^[^ ^38^, ^125^ ^]^ NP morphologies (shapes, sizes, and assembly structures) dictate the plasmon coupling, LSPR, and electric field at the hot‐spot which directly influences the SERS enhancement of the Raman signals. Designing the nanostructure for SERS biomedical diagnosis in bioassays, cells, as well as clinics, have been reviewed in detail in some of the recent articles.^[^ ^126^, ^127^ ^]^

One of the early successful studies using SERS in vivo demonstrated use of labeled SERS gold nanostructures, as shown in Figure 8D, where tumor detection was achieved by spectrally differentiating the SERS signature of the skin and tumors and cross‐referencing them with the label.^[^ ^108^, ^128^, ^129^ ^]^ SERS benefits from the potential for multiplexed detection when using multiple labels, due to the sharp well‐defined spectral peaks. Ou and co‐workers^[^ ^130^ ^]^ tracked SERS of duplexed 4‐(2‐hydroxyethyl)‐1‐piperazineethanesulfonic acid HEPES‐reduced gold nanostars (50–70 nm size) and observed a maximum accumulation of gold nanostars occurring 6 h postintravenous (IV) delivery. Monitoring the 1325 and 1580 cm^−1^ Raman shifted peaks for the SERS tag DTNB (targeting PD‐L1) and pMBA (targeting epidermal growth factor receptor (EGFR)), respectively, both in vivo and ex vivo duplexed detection was achieved.

Recent advances toward the possibility of SERS for use in humans came from the demonstration by Stone et al. of multiplexed deep tissue imaging and detection by a spatially offset Raman spectroscopy (SESORS) and transmission Raman spectroscopy (TRS) set‐up with gold core encapsulated with silica shell nanostructures achieving a record detection depth of up to 5 cm from tissues.^[^ ^131^ ^]^ This was followed by a demonstration of functionalized nanoparticle labeling of bone for SESORS^[^ ^132^ ^]^ and for glucose detection in a mouse model.^[^ ^133^ ^]^ Furthermore, Dey and co‐workers^[^ ^134^ ^]^ have designed and employed customized gold nanoassemblies for deep tissue SORS detection have shown improved efficiency when compared to single gold NPs at identical Au concentration and injection depths. Reports employing an endoscopic fiber bundle for SERS detection^[^ ^135^ ^]^ also note the potential for on‐site detection during surgery for detecting tumor location, as well as surgery margins.

The capability of predicting depths of an inclusion (which may be a microcalcification in a tumor or injected SERS NPs accumulated in the tumor, etc.) by employing trained models and algorithms initially by performing signal‐depth corelation studies was demonstrated by the group of Matousek and Stone.^[^ ^136^, ^137^, ^138^ ^]^ Stemming from these initial works, they have recently reported^[^ ^139^ ^]^ the capability of predicting depths of SERS NPs as inclusions only by measuring Raman signals from external surfaces (SORS and TRS^[^ ^140^, ^141^, ^142^ ^]^) and eliminating the need of a priori data from multiple depths, making it truly noninvasive.

Furthermore, it is not only important to be able to measure the Raman or SERS signal from within depths, but also to be able to fully understand the cancer cell/tissue environment. This would be possible if there were a way to measure its local environment like pH or temperature. It is reported that the cell/tissue pH is significantly different between normal and cancer cells.^[^ ^143^, ^144^ ^]^ Jamieson et al. demonstrated the possibility of measuring subsurface pH levels using SERS (NPs) from zones within cell culture spheroids at depths of 0.5–1 mm using conventional Raman microscopy.^[^ ^145^ ^]^ Gardner et al.^[^ ^146^ ^]^ have reported the detection of pH in the local environment of the NP noninvasively at depth in the pH range of 2–10 and have referred to this as pH‐SESORS. Methyl benzoic acid (MBA) has been employed as the pH‐sensitive SERS tag, tagged onto 100 nm gold NPs. A change in pH resulted in Raman (Stokes) signal shift in the benzene ring stretching mode of MBA with pH.

An even more significant benefit of using SESORS may come from the direct measurement of the local temperature at the surface of the NPs as well as that in the bulk tissue. This would be highly beneficial in tailoring the photothermal therapy for optimum outcomes. Building on an earlier work of Van Duyne's team demonstrating the ability to measure the temperature of individual SERS NPs^[^ ^147^ ^]^ the group of Matousek and Stone have employed the concept of measuring the Anti‐Stokes to Stokes ratio (peak intensity) as a function of temperature and thereby being able to predict the temperature of the Raman signal generating component (matrix or NPs, etc.) noninvasively at depth at its local environment, for the first time. They have demonstrated the concept by measuring signals from Polytetrafluoroethylene (PTFE) (T‐SORS)^[^ ^148^ ^]^ and SERS‐tagged gold NPs (T‐SESORS) inside biological tissue.^[^ ^149^ ^]^ Single AuNPs with a SERS tag were used as the temperature reporters, whereas Au nanoshells acted as heat generators (where Laser illumination wavelength = LSPR wavelength of heat‐generating nanoshells ≠ LSPR wavelength of temperature reporter AuNPs). They measured a maximum temperature rise of 20 °C with the presence of the heat generating nanoshells, solely by measuring the ratio of Anti‐Stokes/Stokes ratio of the SERS tagged gold NPs temperature reporter. Such advances in methodology development help to advance the Raman biodiagnostics technology toward real‐life applications.

#### 2.5.5 Others Potential Optical Techniques

Modified gold nanostructures have very recently been utilized in short wavelength infrared (SWIR, *λ* = 1–2 µm) where they benefit from its photoluminescence quantum yield of about 3.8% at 900 nm.^[^ ^150^ ^]^ Alternative approaches to access signals using longer wavelengths have been explored by the Graham and Faulds group in utilizing chalcogenide labels to provide extreme redshifted SERS nanotags at around 1280 nm.^[^ ^151^ ^]^

Another interesting technique recently reported employed the absorption of the nanostructures (especially, gold NRs) which would therefore reduce the transmission through the tissues to enable detection of the regions accumulating gold NRs. This approach is referred to as SPR enhanced optical imaging/tomography (SPROI/SPROT)^[^ ^152^ ^]^ and it was suggested that this approach leads to improved detection when compared with X‐ray computed tomography. Gold nanostructures have also found applications in OCT.^[^ ^153^ ^]^ To this end, gold nanoclusters^[^ ^154^ ^]^ and gold nanoprisms^[^ ^155^ ^]^ have been explored quite recently.

#### 2.5.6 Multimodal Optical Diagnosis

As in most scenarios, it becomes evident that a single diagnostic modality does not fulfil all the requirements, there has been a significant drive toward combining multiple imaging modalities by either utilizing different properties of an imaging agent or utilizing multifunctional nanostructures to achieve multiple diagnosis in a single platform. Here we will cite some relevant work employing gold nanostructures as a multimodal optical diagnostic agent. Jokerst et al.^[^ ^113^ ^]^ demonstrated the combination of SERS and PA imaging with gold NRs which are also effective photothermal agents. The group envisions the use of PA for characterizing the tumor shape and morphology and the use of SERS for detecting tumor margins during surgery to check for complete resection. Li and co‐workers^[^ ^156^ ^]^ have reported PA–Ultrasound–X ray imaging using modified gold nanostars coated with silica shell. Additionally, thermal imaging was studied for these constructs in vivo. A study by Liu et al.^[^ ^102^ ^]^ featured the use of gold nanostars for SERS, X‐ray, and two‐photon luminescence imaging along with photothermal therapy. They added a fourth modality of MRI diagnosis by attaching gadolinium to gold nanostars and have demonstrated it in tissue phantoms and cells.^[^ ^157^ ^]^ Many such combinations have been suggested with various merits. It is worth reflecting that multimodal approaches, that although there are benefits from multiple imaging techniques, a synergistic balance between them is required to make it successful in research, technology integration, commercialization, and market adoption phases.

### 2.6 Laser Exposure in Laser‐Utilized Theranostics

Both diagnostic and therapeutic applications require laser light sources that when used alone (without NPs present) should not damage healthy tissues. This demands relatively low laser illumination intensities for safety. The maximum permissible exposure to which eye or skin can be accidentally exposed to light (referred to as MPE) defined as one‐tenth of the damage threshold resulting from photothermal and photochemical effects, is a way to quantify the risk of optical radiation exposure.^[^ ^158^ ^]^ Usually, MPE is expressed in irradiance (W cm^−2^) as power spread over a circular aperture. To evaluate the laser irradiance for the purposes of the laser safety standard, one uses the actual illumination area when the area is larger than the so‐called limiting aperture defined by the standard. For the smaller beam areas than the limiting aperture one uses the limiting aperture area itself. MPE and limiting aperture are defined in the IEC 60825‐1 depending on pulse width or exposure duration and the spectral region for both ocular and skin exposure. For example, for exposure time of > 10 s and a wavelength for *λ* = 400–1400 nm, the limiting aperture for both eye and skin is 3.5 mm, while the MPE is 2CA W cm^−2^. As the empirical coefficient CA (also referred as correction factor) is 1 for *λ* = 400–700 nm and increases to 5 for *λ* = 1050–1400 nm. Therefore, the power density employed should be within 2–10 W cm^−2^ depending on the laser line used. Moreover, for specific contact application to nonocular tissue, the irradiance exposure level for the specific treatment procedure may exceed the MPE, e.g., class 1C subject to adhering the defined conditions.^[^ ^158^ ^]^ A clear cost‐benefit analysis and risk assessment should be undertaken prior to considering higher exposure levels, but those much higher than the MPE may be viable in clinical theranostics, particularly when considering the alternative of significant radiation doses and the toxicity from chemotherapy for contrasting risks.

### 2.7 Surface‐Functionalization of Gold Nanostructures

The surface functionalization plays a crucial role, as it is this that controls the interaction with biological components assuring biocompatibility, longer blood‐circulation time, active‐targeting to the cells, and tumor.^[^ ^159^ ^]^ However, this is not in the scope of the current review, we would like to briefly summarize the area and direct the readers to more relevant specific reviews focusing on various aspects of biofunctionalization for applications in nanomedicine. Recent reports and reviews discuss the role of gold nanostructure size and shape,^[^ ^159^, ^160^, ^161^ ^]^ surface charge,^[^ ^68^, ^81^, ^162^, ^163^, ^164^ ^]^ passive targeting,^[^ ^165^ ^]^ active targeting,^[^ ^166^ ^]^ as well as in vivo distribution.^[^ ^167^, ^168^ ^]^ It has been pointed out that a positive surface charge like cetyltrimethylammonium bromide (CTAB) molecules on gold NRs can have a detrimental effect by increasing the toxicity caused to normal cells, but has demonstrated improved uptake by tumor cells.^[^ ^169^ ^]^ Whereas numerous studies suggest that PEG‐based polymers^[^ ^170^, ^171^ ^]^ have been able to provide a neutral surface charge, as well as promote biocompatibility, stealthing (hiding from the immune system), and improving blood circulation times, assuring better chances of uptake by the tumor cells. Once the NPs are injected and are not cleared out by a rapid immune response, they need to reach the specific target locations which can be achieved by either passive targeting or active targeting. Passive targeting utilizes the EPR effect where the nanostructures enter the leaky blood vessels of the tumor via EPR and is not released out of the tumor due to the enhanced retention properties of the tumors. This phenomena has been discussed in Section 3.^[^ ^160^, ^165^ ^]^ Active targeting of tumors requires surface functionalization of gold nanostructures with complementary ligands to those over‐expressed in tumor cells. Typically, antibodies, DNA have been employed, whereas recent aptamers, microRNAs and macrophages are also being utilized for this purpose.^[^ ^160^, ^165^, ^166^, ^168^, ^172^, ^173^, ^174^ ^]^ It is important to note that the targeting ligands should be on the outer corona of the nanostructures, making their binding sites available to the target along with the stealth component, but additionally should not compromise the effectiveness of the theranostics of the nanostructures.

## 3 Toxic Potential, Cellular Uptake, and Biodistribution of Gold Nanostructures

AuNPs have gained much deserved recognition and attention in the field of disease management, especially during the past two decades.^[^ ^175^ ^]^ At the same time, the growing use of AuNPs to better understand their potential toxicity toward healthy cells/tissues into a living system (either at the cellular level or at the level of the whole organism) requires noteworthy concentration to validate their use to solve real‐world clinical problems.^[^ ^176^ ^]^ Around 85–90% of cancers form in epithelial cells, those that line organs and interact most with the ingested toxins and the environment. Healthy epithelial cells are attached to the basal membrane by tight junctions which form a continuous belt around the circumference of each cell.^[^ ^177^ ^]^ NPs can cross cellular membranes via two distinct pathways: the transcellular pathways and paracellular pathways.^[^ ^178^ ^]^ Tight junctions regulate the paracellular exchange of small ions and molecules between cells based on their shape and size; in contrast, cell surface receptors stimulate intracellular passage of ions, which is mainly mediated by ligand binding and enzyme/protein interaction.^[^ ^179^ ^]^ NPs have a tendency to enter injured or diseased cells because of their loose vasculature—this effect is known as EPR effect and is mainly exploited for the accumulation of NPs in tumors.^[^ ^180^ ^]^ Despite recent advancements in targeted nanoformulations, the consideration of off‐target toxic impacts, and metabolism in cellular, endosomal, and lysosomal conditions of AuNPs remain largely unknown. The toxicology patterns among different structures of gold nanostructures make their direct comparison unreliable, which is the result of considerably different physiochemical structures of their bulk and engineered counterparts.^[^ ^181^ ^]^ Furthermore, their cell/tissue‐dependent clearance and cellular uptake differ between AuNPs of different size, shape, compositions, and surface charge.^[^ ^182^ ^]^ However, current knowledge on the toxicological implications and bioavailability of AuNPs has major uncertainties surrounding the fate and behavior of AuNPs in living systems.^[^ ^183^ ^]^ Recently available literature reports conflicting results on the toxicology of AuNPs because of the variety of available preparation methods, functionalization routes, exposure environments/conditions, administration routes, and assessments criteria. Another cause of apparently conflicting results is the variability of used assays for toxicity, cell culture, animal model, dosing parameters, and toxicity evaluation at acute, subacute, chronic, and subchronic levels.^[^ ^180^, ^184^ ^]^

Although pristine gold is generally considered as biologically inert, chemically stable, biocompatible, and nontoxic,^[^ ^185^ ^]^ considerable unwanted toxic effects of AuNPs arise from their variable synthesis routes, size, shape, surface charge, surface conjugates, exposure environments, and administration routes, which are described in Sections 3.1–3.3. Furthermore, there is considerable variance in the ability of AuNPs to interact with cell surface membrane, their cellular uptake and localization with regard to surface modification, coating, size, and shape. Understanding the mechanism of cellular uptake of functionalized and coated AuNPs require characterization prior to their interaction with cells and organs. Coating of AuNPs with CTAB has been widely studied.^[^ ^169^ ^]^ CTAB is a cationic micellar surfactant. CTAB‐coated AuNPs maintain stable dispersions in aqueous solutions if prepared under correct concentrations and ratios. It has also been reported that the higher concentrations of CTAB induces high levels of toxicity.^[^ ^169^ ^]^ Cellular uptake is significantly important for specific and selective targeting of tumors. Nonspecific cellular uptake of AuNPs can induce collateral damages to healthy cells when photothermal treatments are applied. Therefore, the removal of CTAB or such coating agents needs to be ensured in order to achieve specific and selective delivery of NPs to diseased cells.^[^ ^186^ ^]^ Recent in vitro and in vivo toxic effects of surface coated and pristine AuNPs have been described and compared in **Table** **2**.

**Table 2:** In vitro and in vivo toxicity of AuNPs

Nanoparticles/composites	Size	Surface coating	Dosage rate and exposure time	Model (cells/animals)	Comments on toxicity (effect on cells, organs, tissues/cell viability)	References
Nanospheres	2 nm	—	0.38–3 × 10^−6^ m; 1, 2.5, 6, 24 h	COS‐1	Cationic particles are moderately toxic, whereas anionic particles are nontoxic facilitated by their strong electrostatic attraction to the negatively charged bilayer	^[^ ^229^ ^]^
Nanorods	4 nm	Chitosan on the surface	50 µg mL^−1^, 24 h	Mice	Improved in vitro cellular uptake and minimal toxic effects were observed	^[^ ^230^ ^]^
Bifunctional Au/Ni NRs	20 µm long and 170 nm in diameter	—	44 mg mL^−1^ 4 h	HEK293	Reduced risk of cytotoxicity and immunogenicity.	^[^ ^231^ ^]^
Nanospheres	3.5 ± 0.7 nm	—	10, 25, 50, and 100 × 10^−6^ m 24, 48, and 72 h	RAW264.7	Au NPs are not cytotoxic, reduce the production of reactive oxygen and nitrite species, and do not elicit secretion of proinflammatory cytokines TNF‐*α* and IL1‐*β*, making them suitable candidates for nanomedicine.	^[^ ^232^ ^]^
Nanospheres	2, 10, 25, 40, 50, 70, 80, and 90 nm	Herceptin physical adsorption	10 µg mL^−1^ 3 h	SK‐BR‐3, SNB‐19, and HeLa cells	Gold and silver NPs coated with antibodies can regulate the process of membrane receptor internalization	^[^ ^233^ ^]^
Nanoshperes	50 and 100 nm	Tiopronin	1 nmol L^−1^ 3–24 h	MCF‐7	Optimal smaller size for NPs that maximizes their effective accumulation in tumor tissue.	^[^ ^234^ ^]^
Nanospheres	4, 12, and 17 nm	L‐cysteine	10 × 10^−9^ m 3 h	HeLa	Both the uptake and unbinding force values are dependent upon the size of gold NPs.	^[^ ^235^ ^]^
Nanorods		CTAB, PEG‐SH	0.01–0.5 × 10^−3^ m 24 h: In vitro (0.5–0.9 × 10^−3^ m in vivo) 0.5, 3, 6, 12, 24, and 72 h: In vivo	HeLa/mice	PEG‐modified gold NPs showed a nearly neutral surface and had little cytotoxicity in vitro. Following intravenous injection into mice, 54% of injected PEG‐modified gold NPs were found in blood at 0.5 h after intravenous injection, whereas most of gold was detected in the liver in the case of original gold NRs stabilized with CTAB.	^[^ ^236^ ^]^
Nanostars	110 10 nm	GNS SiO2/Au	10 mg g^−1^ 4 h, 1, 4, 7, 14, 21, 28 d	Mice IV;	The mass of gold in the tissue samples ranged from our determination limit (about 70 pg) to a few micrograms.	^[^ ^237^ ^]^
Nanowires	0.58, 1.8, 4.5, 8.6 nm_X 200 nm	Thiols with amino, alkyl, or carboxyl end groups, serum	103–106 particles mL^−1^, 24 h	NIH 3T3	Internalized nanowires with high aspect ratios are more toxic to cells than nanowires with low aspect ratios.	^[^ ^238^ ^]^
Nanoclusters	0.8, 1.2, 1.4, 1.8, CG‐15	Triphenylphosphine	1–10 000 × 10^−6^ m 6, 12, 18, 24 h	HeLa Sk‐Mel‐28 L929 J774A1	Gold particles 15 nm in size and Tauredon (gold thiomalate) are nontoxic at up to 60‐fold and 100‐fold higher concentrations, respectively. The cellular response is size‐dependent, in that 1.4 nm particles cause predominantly rapid cell death by necrosis within 12 h while closely related particles 1.2 nm in diameter effect predominantly programmed cell death by apoptosis.	^[^ ^194^ ^]^
Nanoclsuters	1.4 nm	—	Mice: 57 mg, Rat: 285 mg Mice: 2, 4, 24 h Rat: 3, 7, 10 d	Mice, Rat	clusters reach a polydentate ligand sphere that increases the kinetic stability by orders of magnitude	^[^ ^195^ ^]^
Nanoparticles	12.5 nm	—	IP; 40, 200, 400 mg kg^−1^ day^−1^ 8 d	Mice	AuNPs are able to cross the blood–brain barrier and accumulate in the neural tissue. Importantly, no evidence of toxicity was observed in any of the diverse studies performed, including survival, behavior, animal weight, organ morphology, blood biochemistry and tissue histology.	^[^ ^239^ ^]^

### 3.1 Impact of Particle Size and Shape

The key parameter determining physiochemical properties of AuNPs is their size, which strongly influences the in vitro and in vivo behavior of the theranostic platform. The geometric effects of their clusters and their size order constitute crucial parameters which control their biodistribution, cellular uptake, endocytosis effectiveness, and clearance sites and clearance rates.^[^ ^187^ ^]^ It has been reported that pristine surfaces of NPs with a diameter less than 100 nm may be able to enter cells,^[^ ^182^ ^]^ while similar pristine NPs smaller than 40 nm in diameter may approach the cellular nuclei.^[^ ^187^ ^]^ NPs smaller than 35 nm in diameter can reach the brain by crossing the blood–brain barrier, while NPs in diameter less than ≈10 nm are excreted from the body via renal filtration.^[^ ^188^ ^]^ Therefore, the targeted delivery of gold nanostructures in the tumor rely on size and surface functionalization/coating, which in turn can facilitate optimum theranostic activity along with minimum side‐effects and toxicity toward healthy cells/tissues.^[^ ^182^ ^]^ The extent of potential entry and clearance pathways of NPs can be changed/directed by using functionalizing/coating agents which affect the penetration/transport of NPs to cell membrane. Considerable work has been carried out to investigate the particle‐shape and surface functionalization‐dependent accumulation and delivery of gold nanostructures and their corresponding toxicological effects on different in vitro and in vivo test models.

**Figure 9:**
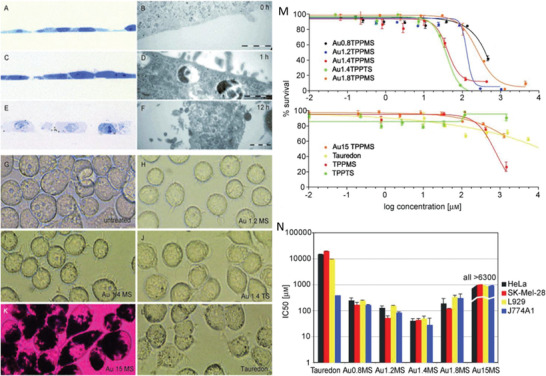
Microscopic images of HeLa (A–F) and J774A1 cells (G–L) treated with Au nanostructures. A–F) HeLa cells were treated with gold nanoclusters over 0, 1, or 12 h time. Cells were fixed, stained, and seen with an optical microscope (A,C,E) and with and scanning electron microscope (B,D,F). G–L) J774A1 macrophages were treated for 1 h and imaged using an optical microscope. AuNPs of 15 nm diameter stained the endocytic compartment of the cells black sparing the nucleus (K). Cytotoxicity of AuNPs against four cell lines (HeLa, SK‐Mel‐28, L929, and J774A1). M) HeLa cells were seeded at 2000 cells per well and were treated with AuNPs for 48 h and MTT tests were carried out for the evaluation of cell viability. N) IC_50_ values of Au 1.4MS were lowest across all cell lines and that AuNPs of smaller or larger size were progressively less cytotoxic, revealing a size dependent cytotoxicity. Adapted with permission.^[^ ^194^ ^]^ Copyright 2007, Wiley.

**Figure 10:**
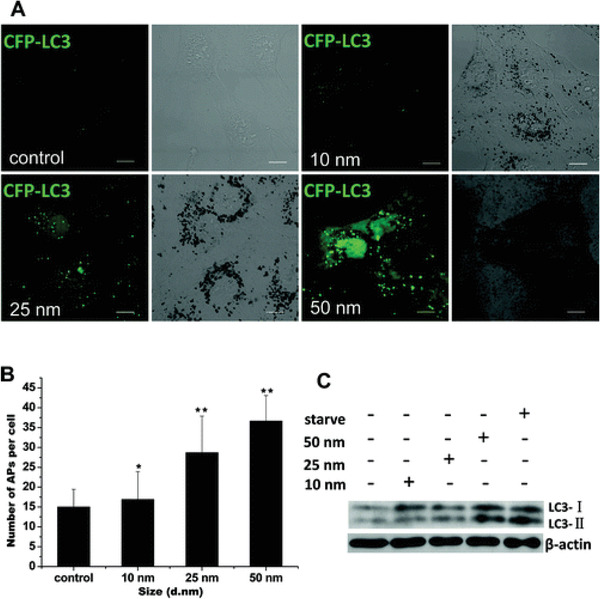
Induction of LC3 puncta by AuNP treatment. A) Formation of cyan fluorescent protein (CFP)‐tagged LC3 (LC3‐CFP) (pseudocolored as green) in CFP‐LC3 normal rat kidney (NRK) cells treated for 24 h with 1 × 10^−9^ m AuNPs. Left, confocal image; right, bright‐field image (scale bar, 10 µm). B) Statistical analysis of the number of autophagosomes per cell after 24 h of treatment. C) Conversion of LC3 from the cytoplasmic form (LC‐I) to the autophagosome‐associated form (LC3‐II). Adapted with permission.^[^ ^197^ ^]^ Copyright 2011, American Chemical Society.

AuNPs have long been considered to be nontoxic.^[^ ^189^, ^190^, ^191^ ^]^ However, various cytotoxic effects have been described in a size and shape‐dependent manner. The potential causes of toxicity of AuNPs are the release of cytotoxic ions/radicals, and translocation across the cell membrane into mitochondria. Most importantly, internalization of NPs into cells, the modification of cellular signaling pathways, and destruction of cells/cell membrane can be other sources of toxicity.^[^ ^192^ ^]^ Cellular uptake of AuNPs is greatly influenced by nonspecific adsorption of proteins from the serum onto the surface of NPs, which can increase their attachment to the cell membrane and may induce receptor‐mediated endocytosis of AuNPs.^[^ ^193^ ^]^ Pan et al.^[^ ^194^ ^]^ reported cytotoxicity of a series of AuNPs ranging in size from 0.8 to 15 nm against SK‐Mel‐28, HeLa human cervix carcinoma, L929 mouse fibroblasts, and J774A1 mouse macrophages and observed that cellular uptake of AuNPs was directly dependent on particle size. NPs ranging from 1 to 2 nm in diameter were highly toxic, while both smaller and larger NPs were nontoxic. NPs of size 1.4 nm induced cell death by necrosis, while NPs of size 1.2 nm caused programmed cell death by apoptosis. Size‐dependent toxicity of AuNPs is illustrated in **Figure** **9**. In another study, Tsoli et al.^[^ ^195^, ^196^ ^]^ reported the toxicity of bare Au_55_ clusters (isomers of a 55‐atom gold cluster) of 1.4 nm (roughly the size of DNA) revealed strong interaction between NPs and grooves of DNA showing good biocompatibility and reduced toxicity. Ma et al.^[^ ^197^ ^]^ found that AuNPs of 10, 25, and 50 nm in diameter were uptaken by normal rat kidney cells via endocytosis in a size‐dependent manner, which is likely related to the accumulation of NPs in lysosomes resulting in lysosome degradation via alkalinization of endocytic essentials. AuNPs induced accumulation of LC3‐positive punctuate structures when treated with normal rat kidney (NRK) cells which stably expresses cyan fluorescent protein (CFP)‐tagged LC3 (LC3‐CFP) (see **Figure** **10**). In another study, Coradeghini et al.^[^ ^198^ ^]^ established that AuNPs with diameter of 5 exhibited toxicity in Balb/3T3 mouse fibroblasts with decrease in cell viability from 47.0% to 36.6%, 13.6%, and 6.3% at the exposure time of 2, 24, and 72 h, respectively. Toxicity was only observed for 5 nm NPs at concentration ≥ 50 × 10^−6^ m leading to their internalization within intracellular endosomal compartments.

Particle diameter and surface charge are key factors determining uptake of NPs into cells. For example, Him et al.^[^ ^199^ ^]^ described the administration of negatively charged AuNPs ranging from 1.4 to 200 nm in diameter and positively charged 2.8 nm in diameter into rats. Accumulation of negatively charged 1.4 and 200 nm sized AuNPs in the liver increased from 50% to 99%, respectively. While positively charged 2.8 nm NPs led to considerably dissimilar accumulations in several organs in comparison to negatively charged NPs of similar size.

**Figure 11:**
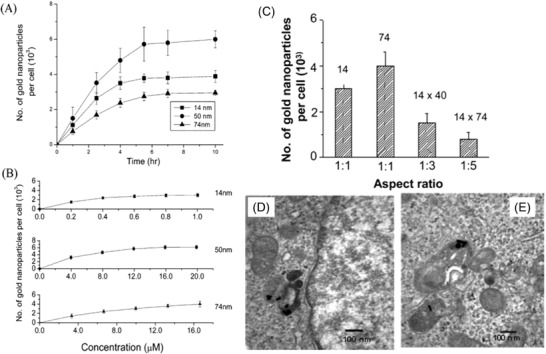
Cellular uptake kinetics of AuNPs. A) Cellular uptake of AuNPs (14, 50, and 74 nm) as a function of incubation time. B) Dependence of cellular uptake of AuNPs as a function of concentration. C) Comparison of uptake of rod‐shaped NPs and spherical shaped NPs (with aspect ratio 1:3 and 1:5), D,E) the transmission electron microscopy images of rod‐shaped NPs (D—1:3, E—1:5) internalized within Hela cells. Adapted with permission.^[^ ^202^ ^]^ Copyright 2006, American Chemical Society.

The shape of AuNP affects its surface area, its surface motion, surface plasmon shift, surface energy, ligand length, and binding distance, which in turn affects its deposition, translocation, distribution, binding energies, membrane binding energies, uptake, fate, and bioavailability in cells, tissues, and organs.^[^ ^61^, ^200^, ^201^, ^202^ ^]^ **Figure** **11** shows that cellular uptake of rod‐shaped NPs occurs slowly as compared to spherical‐shaped NPs. It is likely that the different levels of internalization of NRs and nanospheres are influenced by their shape and surface morphology. In another study, Zhang et al.^[^ ^203^ ^]^ presented the biodistribution of PEG‐coated AuNPs in mice, which did not cause toxicity. Specifically, 5–10 and 30 nm AuNPs accumulated in the liver and spleen of mice, respectively, while 60 nm NPs aggregated preferentially in the blood cells.

### 3.2 Impact of Surface Chemistry (Surface Functionalization)

The exterior surface of AuNPs can be functionalized and modified with small biomolecules, peptides, ligands, antibodies, and their fragments and nucleic acid to produce specific targeting and biocompatibility of AuNPs for therapeutic, diagnostic, and drug delivery applications.^[^ ^204^, ^205^, ^206^ ^]^ However, the role of such functionalization alterations play in determining the cellular toxicity of AuNPs is still not well understood. Therefore, it is undoubtedly important to evaluate the relationship between functionalization/bioconjugation and cellular uptake/toxicity. Hauck et al.^[^ ^207^ ^]^ revealed that use of polyelectrolyte as functionalizing agent can be used to improve the uptake of gold NRs to HeLa cells by modifying the surface chemistry of NPs using polyelectrolyte. The cell viability measured in most of the experimental parameters were higher than 90% even at high concentrations. The expression levels of oxidative stress, such as heat‐shock protein or protein activity did not exhibit up‐ or down‐regulation following rod‐like AuNPs, which reveals that functionalized NPs did not induce noticeable toxicity.

**Figure 12:**
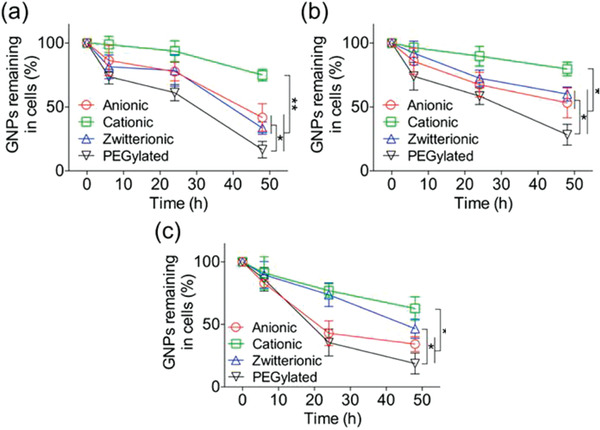
Exocytosis of serum‐coated AuNPs in macrophages. A–C) Exocytosis rate of serum‐coated AuNPs with size ranges of 10 (A), 20 (B), and 40 nm (C) in macrophages when treated NPs for 6, 24, and 48 h. Adapted with permission.^[^ ^208^ ^]^ Copyright 2014, American Chemical Society.

Little attention has been paid on the effect of functionalization/bioconjugation of AuNPs on the excretion and exocytosis of AuNPs when they leave macrophages. Recently, Oh et al.^[^ ^208^ ^]^ showed that the bare and functionalized AuNPs mediate their exocytosis patterns in macrophages. Cationic NPs were found to be retained in the cells, while PEGylated NPs are likely to be transferred in the cytoplasm, which in turn clear the NPs due to the interactions of the NPs with intracellular proteins, trafficking, and signaling pathways.^[^ ^208^ ^]^ **Figure** **12** shows the exocytosis of serum‐coated AuNPs in macrophages with size ranges of 10 A), 20 B), and 40 nm C). Chandran et al.^[^ ^209^ ^]^ examined the effects of bare and protein/corona functionalized AuNPs ranging from 40 to 80 nm in diameter on corona composition and its ultimate effect on cellular uptake, toxicity, and gene expression responses in human umbilical vein endothelial cells. The findings suggested that the cellular uptake of bare NPs was reduced as a result of plasma corona formation. AuNPs are rapidly covered by a selective group of biomolecules when they enter a biological environment and form a corona due to the negatively charged AuNP and positively charged proteins. Corona formation alters the physical and chemical properties of NPs, which in turn can cause aggregation via electrostatic interaction. Consequently, aggregation affects the ability of NPs to enter target cells. The negatively charged surface of AuNPs potentially bind with positively charged extracellular proteins, which in turn reduces their ability to be up taken by cells, while positively charged AuNPs were easily transported into cells due to the electrostatic interaction with negatively charged cell membrane. This type of interaction of NPs with cell membranes can break the cell membrane. Based on the results discussed earlier, it can conclude that surface charge, the interaction of NPs with proteins and the cell membrane play a critical role in cellular localization and internalization.^[^ ^199^ ^]^

Therefore, surface modification of AuNPs with appropriate ligands and concentrations proved to greatly affect the extra‐ and intracellular fates of NPs.

### 3.3 NPs Administration Routes

Biodistribution, cellular uptake, internalization, accumulation, and clearance of NPs clearly depends on administration route in addition to particle size and shape.^[^ ^201^ ^]^ It has been demonstrated that AuNPs smaller than 5–15 nm have more target cellular uptake than larger NPs of 50–100 or above.^[^ ^207^, ^209^ ^]^ Liver and spleen are the main organs of living systems which have shown increased accumulation of NPs with the increase in AuNP diameter.^[^ ^210^ ^]^ AuNPs have been found to have long blood circulation times when their diameter increased.

**Figure 13:**
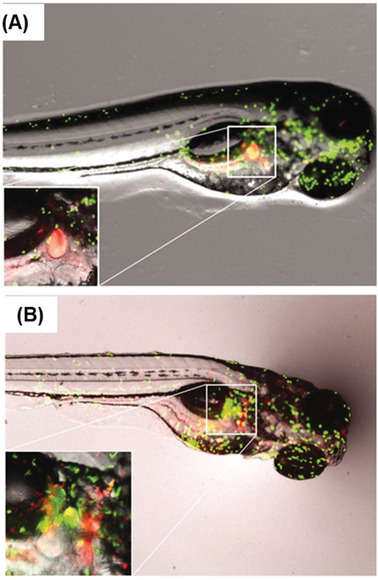
A) Images of fluorescent neutrophils and macrophages in zebrafish embryos after exposure to gold nanourchins. The inset is providing further details using 2 × magnification. B) Images of fluorescent neutrophils and macrophages in zebrafish embryos after exposure to gold nanobipyramids. The inset is providing further details using 6 × magnification. Adapted with permission.^[^ ^210^ ^]^ Copyright 2019, Taylor & Francis.

Moreover, in vivo studies demonstrated that the administration route impacts the toxic effects in living systems. There are a variety of administration routes available for the introduction of NPs to the living systems, such as oral, intravenous, intra‐tumor, oral, intraperitoneal, and tail vein. Fan et al.^[^ ^211^ ^]^ examined the effect of three administration routes, including oral, intraperitoneal, and tail vein injection, on toxicity of AuNPs of 13.5 nm, revealing that oral and intraperitoneal injections caused the highest toxicity, while tail vein injections showed the minimal toxicity, which suggested that tail vein injections could potentially be an appropriate route for targeted AuNPs. Tail vein and oral administration impact most negatively on body weight particularly after 6 and 12 days, respectively, when compared to the control. Selection of administration route may change the time required to reach the target, side effects, enzyme activity based on nutritional status, and dilution of NPs. Because different types and sizes of NPs generate different actions when given by different administrations routes. The underlying mechanisms involved in the effects of administration routes largely remains unknown. Jong et al.^[^ ^212^ ^]^ evaluated the biodistribution of AuNPs (of 10, 50, 100 m and 250 nm in diameter) in intravenously injected rats suggesting the size‐dependent distribution of NPs. This study revealed that most NPs were found in the liver and spleen, while the smallest 10 nm NPs were detected in the blood, liver, and spleen. It has also been found that macrophages were found to take AuNPs from the tissue fluid, transfer them into the veins and then be transported to digestive organs for clearance.^[^ ^210^ ^]^ The shape‐dependent biodistribution of AuNPs, into the main digestive organs in zebra fish, showed a high abundance of immune cells in the gallbladder (**Figure** **13**A), pancreas, and/or liver (Figure 13B). Nanospheres were transported to the liver while nanourchins were predominantly found in the gallbladder. A macrophage loaded with AuNPs can be seen in the tissue (confirmed by the fluorescent signal). AuNPs thereafter were transported to the liver or spleen to be cleared from the body.

AuNPs with controlled size, shape, composition, and structure for their selective delivery to target sites raise important safety constraints for application in cancer nanotheranostics. Despite recent developments in the synthesis of gold nanostructures, a complete elucidation of the mechanisms of AuNP cellular localization, biodistribution, interplay between surface functionality, biological interactions, and their interaction with microenvironmental fluid is still a major challenge. In cancer nanotheranostics, the key driver is selective delivery and retention of NPs by tuning the physicochemical properties of NPs in enhancing theranostic efficacy while remaining nontoxic toward healthy surrounding tissues. Based on nanomedicine approaches, the biological fate of NPs depends on three factors; first, physicochemical features of NPs including size, shape, surface charge, light‐triggered effects, and functionalization/bioconjugation; second, biological factors, such as microenvironmental fluid, extracellular proteins, redox signaling molecules, and cellular activation; and third, experimental factors, such as temperature (incubation and hyperthermia) and pH of media. These issues have already been discussed in detail in several excellent reviews.^[^ ^60^, ^61^, ^213^, ^214^, ^215^ ^]^

The internalization of AuNPs is more likely to occur via endocytosis, which leads to engulfment of extracellular fluid and NPs via plasma membrane ruffling followed by membrane wrapping of NPs and cellular uptake.^[^ ^216^ ^]^ The other process involved in the transport of NPs into cells is direct diffusion via cellular membranes. There are five main types of endocytosis: phagocytosis, clathrin‐mediated endocytosis, caveolin‐mediated endocytosis, clathrin/caveolae‐independent endocytosis, and micropinocytosis.^[^ ^217^ ^]^ The process of phagocytosis is mediated by the binding of receptors present in the membranes of phagocytes. Pinocytosis involves the internalization of NPs via small pinocytic vesicles. The abundance of caveolae (a type of lipid raft, and are small invaginations of the plasma membrane) in cells impacts the potential of NPs for caveolae‐mediated endocytosis, while a clathrin‐binding protein attached to proteins at membranes mediates the formation of clathrin‐coated vesicles, which help transport NPs into cells.^[^ ^218^ ^]^ Direct translocation or endocytosis‐mediated entry of AuNPs through cell membranes results in the disruption of the lipid bilayer integrity, which usually results in cellular toxicity. Endocytosis is mainly considered the major process involved in the entry pathways of NPs. Few other processes have also been demonstrated, such as passive pathways (diffusion), disruption of lipid bilayers, and nanoscale hole formation, and microinjection/electroporation techniques.^[^ ^219^ ^]^ The effects of other key factors including composition, size, shape, surface charge, surface functionalization, and surface hydrophobicity/hydrophilicity, and their interactions with cells have been discussed earlier in this section.

The notable cellular fate/transcellular trafficking features, cellular uptake, and cellular localization of AuNPs have mainly been revealed by imaging and chemical analysis tools. These techniques include scanning and transmission electron microscopies, atomic force microscopy, scanning tunneling microscopy, flow cytometry, Raman spectroscopy, photoacoustic microscopy, photoablation ICP‐MS, dark‐field microscopy, and super‐resolution fluorescence microscopy. These approaches provide fundamentally derived experimental evidence capable of describing specific spatial precision at nanoscale and localization of NPs to analyze the binding site distribution of NPs at the cellular level, as well as specific binding and molecular tagging. Each technique has its own merits and disadvantages to achieve specific biomolecular information from samples.

AuNPs, being a noble metal, displays specific optical properties which can be used as sensing elements with excellent stability of Raman tags, since it is easy to prepare and provides favorable light scattering characteristics. Coherent anti‐Stokes Raman scattering has recently been used for the cellular localization and imaging of uptake of NPs.^[^ ^220^, ^221^, ^222^, ^223^ ^]^ In another study, SERS tags have been used to image the distribution of NPs.^[^ ^215^ ^]^ Therefore, Raman scattering provides key insights into the microscopic localization of NPs at intracellular levels which can potentially be used for photothermal nanotheranostics.^[^ ^225^, ^226^, ^227^, ^228^ ^]^

## 4 In Vitro and In Vivo Examples of Light‐Mediated Theranostics: Photothermal Therapy with Optical Diagnosis Techniques

Current clinical diagnosis relies heavily on histopathology of biopsies (excised tissues), which is time and labor consuming, inherently subjective and often misinterpreted for heterogeneous tumors, and limited in multiplexed detection of multiple biomarkers in biopsies. More importantly, in vivo early stage diagnosis (without undergoing biopsies) and diagnostic image guided operations for precision cancer removal are not currently a reality. These have been the key driving factors for researchers to develop a diagnostic technique that can cater for the above needs. Furthermore, the possibility of diagnosis and therapy combined into one platform “theranostics” not only allows for early‐stage diagnosis, image‐guided operation, and therapy, but also therapeutic monitoring allowing personalized medicine. Based upon the previous sections of this review it is clear that light‐mediated gold nanostructure theranostics has the potential to become a powerful clinical tool. To that end, we will discuss here some interesting recent research focusing on gold nanostructures, as the primary agent demonstrating theranostics with both optical diagnosis and PTT therapy. Although there has been a multitude of work undertaken either in the area of preparing gold nanostructures, using them for therapy or detection, there has been comparatively limited work featuring in vitro and in vivo applications of gold nanostructures for both.

To date, X‐ray and NIR fluorescence imaging coupled with PTT have been studied less than Raman SERS and PA. Gold NRs with X‐ray imaging chemo‐PTT in vitro.^[^ ^240^ ^]^ Tian et al. report the X‐ray absorption coefficient which suggests that the attenuation of PEG‐amine coated gold nanostars was 3.6‐fold higher than that of the commercial CT contrast agent iodixanol at the same concentration of 25 mg L^−1^.^[^ ^241^ ^]^ They also demonstrated light‐induced cell death, i.e., PTT.

NIR fluorescence has also been employed in combination with PTT. For example, gold nanorod modified with custom‐synthesized acymmetris cyanine was reported for stimuli‐responsive NIR on/off fluorescence imaging. The acymmetris cyanine in its base neutral form is nonfluorescent, whereas, in acidic condition, the N protonates and changes in structure which exhibits fluorescence. This was then employed as a pH‐responsive nanorod probe for fluorescence imaging and light (808 nm) triggered photothermal therapy.^[^ ^242^ ^]^ Among the multimodal approaches, typically a technique with high spatial resolution or tissue depth penetration and relatively low sensitivity (like, X‐ray, PA) has been synergistically combined with highly sensitive and specific techniques otherwise having low spatial resolution or depth penetration (like NIR fluorescence, SERS etc.). MRI is a nonoptical imaging technique with relatively high spatial resolution (mm) in soft tissue which has been often complemented with other imaging techniques by modifying gold structures with iron oxide or gadolinium, etc., in improve the diagnostic efficacy of these core–shell NPs.^[^ ^71^, ^102^, ^243^, ^244^, ^245^ ^]^ Below, we discuss in detail the applications of PTT with SERS or with PA, as there has been growing interest in the field recent.

Some interesting studies demonstrate the use of different gold nanostructures for SERS optical diagnosis coupled with PTT. Chemo‐drug cisplatin‐loaded gap‐enhanced Raman tags have been reported by Zhang et al.^[^ ^246^ ^]^ suggesting that 0.1 × 10^−9^ m concentration and 3 W cm^−2^, 808 nm laser illumination conditions resulted in 100% cell death as compared to 20% cell death without laser irradiation, i.e., without PTT. It was demonstrated that the SKOV3 ovarian cancer cells are most effected by the photothermal effect rather than the chemotherapeutic effect.

Further in vivo intraoperative Raman‐guided chemo‐photothermal therapy has been demonstrated by Zhang and co‐workers^[^ ^246^ ^]^ for advanced ovarian cancers with disseminated microtumors. This suggests the benefits of combining SERS diagnosis and PTT into one platform of designed gold nanostructures to target cancer theranostics. Multiplexing, an important aspect, has been reported by Bhatia and co‐workers^[^ ^130^, ^247^ ^]^ who used gold nanostars for SERS mapping of ex vivo tumor, as well as for in vivo multiplexed SERS diagnosis. They utilize PD1 and EGFR targeted nanostars for the MDA‐MB‐231 breast cancer cell line employing 170 µg mL^−1^ functionalized nanostars and advocate that they can control cell death specifically at the laser‐irradiated zone.

It is understood that any small molecule (like optical tags/labels, etc.) when leaching out into the cell environment can cause an increase in cell toxicity, but on the other hand, sufficiently high concentration of such labels are required to be able to visualize or image them in in vivo conditions. Therefore, there has been a huge drive toward functionalizing gold nanostructures to provide biocompatibility. Polyethylene glycol‐based polymers are the first choice and much of it has been discussed in the cell toxicity section. To this end, polydopamine has also found use and has been reported^[^ ^248^ ^]^ to provide near‐100% cell viability for gold NRs‐coated with SERS tag pMBA, further coated with polydopamine (in concentration ranges of 1–200 µg mL^−1^ of gold), as opposed to dramatically decreased cell viability from 90% to 15% for gold NRs coated only with pMBA. An increase by 5–70 °C after 5 min of NIR laser irradiation was shown in the pure nanostructure colloid. These structures were then demonstrated to be useful for both SERS detection and PTT using the same laser.

Nanostars (≈40–70 nm) have become popular for SERS‐PTT theranostics applications in various reports.^[^ ^101^, ^249^ ^]^ Bhatia and co‐workers^[^ ^104^, ^250^, ^251^ ^]^ have made elaborate contributions in utilizing gold nanostructures especially gold NRs^[^ ^122^, ^252^ ^]^ for SERS diagnosis, as well as PTT. Folic acid functionalized gold nanobipyramids with 2‐naphthaleinthiol as the SERS tag has been reported for SERS diagnosis and PTT of MCF7 breast cancer cell line both for in vitro and in vivo studies.^[^ ^253^ ^]^ Hybrid gold nanostructures, such as silica‐coated SERS labeled gold NRs^[^ ^254^, ^255^ ^]^ have also been investigated.

Song and co‐workers^[^ ^108^ ^]^ suggest that by tailoring a redox active polymer nanoparticle, porous branched gold nanoshell structures can be prepared, which has benefits in remote laser targeted drug release utilizing the photothermal properties of the gold nanostructure and bimodal optical detection of SERS and PA imaging. Carbon‐based nanomaterials, such as carbon nanotube and reduced graphene oxide with light absorption wavelength from UV to NIR region and photothermal conversion through nonradiative decay have also been explored in conjunction with gold nanostructures. Gold nanoshell coated onto carbon nanotube rings has been employed as a multimodal diagnostic agent for SERS‐PA imaging, as well as PTT.^[^ ^256^ ^]^ Graphene oxide wrapped gold nanords^[^ ^257^ ^]^ serve as SERS‐chemo PTT theranostic agent. Dox loaded onto the nanostructures provides additional pH responsive drug release and SERS behavior, thus providing a way to determine whether the Dox‐loaded nanostructures have been engulfed by a tumor cell which is generally acidic in the environment. Another report uses an elaborate construct of reduced graphene oxide sheets with embedded gold NPs in their nanoholes, creating SERS hot‐spots, labeled with Raman tags. Targeting EGFR lung cancer cells (A549) they report high photothermal efficiency with a power density (0.5 W cm^−2^ of 808 nm NIR laser for 5 min) as a result of synergistic effect by conjugated AuNPs and nanosheets employed at a concentration of 100 µg mL^−1^.^[^ ^258^ ^]^ Gold nanostructures have also found applications for PTT therapy, in addition to, bimodal diagnosis, for example, with magnetic iron oxide‐gold nanostructures where the iron oxide supports MRI providing wide area spatial scan for tumor and gold nanostructure supported SERS imaging aids in determining the tumor margins with higher accuracy.^[^ ^164^, ^249^, ^259^ ^]^

Multispectral optoacoustic tomography (MSOT)^[^ ^260^, ^261^ ^]^ has provided PA imaging with potential to become a clinical possibility. NIR absorbing gold NRs have found immense applications in MSOT‐PA imaging and superseded the traditional use of NIR absorbing dyes.^[^ ^262^ ^]^ Gold NRs functionalized with PEG/PEI polymer were successfully employed to provide an in vitro PTT effect on 293T‐GFP cells incubated with PEI‐NRs with 5.0 µg per well and irradiated for 10 min with laser (808 nm, 1 W cm^−2^) and resulted in significant cell death.^[^ ^263^ ^]^ In vivo temperature rises of ≈30 °C was reported for similar laser irradiation as used for in vitro studies. Micro‐RNA bound gold NRs have been reported as both PA and NIR fluorescence agents by incorporating a fluorescent tag onto the gold NRs via RNA hybridization.^[^ ^264^ ^]^ The fluorescence signals in MCF‐7 breast carcinoma and HeLa cervical carcinoma cells treated with the above nanostructures were reported to be enhanced 7‐ and 4.5‐fold, respectively, compared with nonamplified system and a temperature rise of about 22 °C. Furthermore, gold NRs coated with silica shell are excellent candidates for photoacoustic imaging. Xu and co‐workers report such structures PA imaging, as well as combined chemo PTT therapy.^[^ ^265^ ^]^ Nanovesicles of semiconducting polymer‐plasmonic gold nanostructures^[^ ^266^ ^]^ provides a unique way to enhance the ultrasound and PA optical imaging properties, as well as the PTT properties, thanks to the nanovesicle structure promoting plasmonic coupling enhancements. Doxorubicin (Dox)‐loaded nanopores in the silica shell aided chemo drug release utilizing laser irradiation and PTT effect of gold NRs. They suggested 10 mg mL^−1^ of the above NPs showed significant improvement as compared to chemo or PTT alone. The passively targeted NPs provided a temperature rise of ≈60 °C and about 4 times relative PA signal enhancement.

Gold NPs embedded in graphene sheets have also been utilized for PA‐PTT theranostics. Additionally, NIR fluorescence imaging with excitation/emission: 670/690 nm could aid in achieving a multimodal platform. SCC7 tumor‐bearing mice were subjected to exposure time of 10 min with 808 nm laser at 0.75 W cm^−2^ which resulted in ≈16 °C temperature rise and higher PA signals than control group.^[^ ^267^ ^]^ Their study also demonstrates that PA imaging provided better spatial resolution than NIR fluorescence imaging. The study reported by Yan et al.^[^ ^268^ ^]^ also supports the claim that graphene oxide‐gold nanostructures demonstrate significant enhancement in PA imaging and reports a NIR fluorescence‐PA imaging and PDT‐PTT therapy multimodal approach.

X‐ray CT‐PA, Fluorescence‐PA bimodal diagnostics have been exploited with gold nanostructures. You et al.^[^ ^269^ ^]^ report the use of hollow gold nanoshells as PA, X‐ray CT, and PTT agent, along with multimodal imaging comprising of NIR fluorescence agent via Indocyanine green dye and MRI with gadolinium incorporated into the hollow gold nanoshells. Similarly, hollow gold NRs^[^ ^270^ ^]^ with optimized aspect ratio also find applications as PA–X‐ray CT–PTT theranostic agent. Such hollow NRs were synthesized by a Se‐doping Te nanorod‐templated method with the assistance of L‐cysteine. In another report, gold nanoplates are employed for PA–X‐ray CT imaging along with PTT in lung cancer cell lines.^[^ ^271^ ^]^ They prepared anti‐EGFR peptide‐conjugated PEGylated triangular gold nanoplates of dimensions ≈80 nm and employed them in vivo PA–CT imaging and a temperature rise of ≈21 °C. Laser mediated assembly formation of gold NPs and further employing them for PA imaging with a combination of 405 nm and NIR laser, has been achieved by Cheng and co‐workers.^[^ ^272^ ^]^ DNA‐mediated gold nanorod assemblies have proven to be better candidates than the gold NRs itself in cellular uptake, PA imaging, as well as temperature rise of 15 °C due to PTT effects.^[^ ^273^ ^]^ This thereby drives the interest in developing and utilizing gold nanoassemblies in theranostics.

**Table** **3** lists some of the examples of in vivo theranostic applications employing gold nanostructures for optical diagnosis with PTT therapy. It is apparent that sub‐100 nm gold nanostructures have found their way to in vivo experiments, especially in mouse models. These are usually grafted with tumors from human or mice cells, with breast cancer cell type being the most studied.

**Table 3:** Comparison of in vivo applications of combined optical diagnosis and PTT

Theranostic agent	NP size	Diagnosis	In vivo model	Cancer tumor cell type	Remarks	Refs.
Nanostars	50–70 nm	Raman SERS	Mice	Human Breast cancer MDA‐MB‐231	Multiplexed detection	^[^ ^123^ ^]^
Gold NPs embedded in graphene sheets	15 nm NPs formed on graphene sheets	PA imaging and NIR fluorescence	Mice	SCC7 tumor	Spatial resolution achieved was better in PA imaging	^[^ ^267^ ^]^
Triangular gold nanoplates	≈80 nm	PA and X‐ray CT imaging	Mice	HCC827 tumor	Temperature rise of 21 °C was noted	^[^ ^271^ ^]^
Gold–Silver nanotriangles	≈80 nm	PA and Raman imaging	Mice	MGC 803	Hybrid gold–silver nanostructure	^[^ ^274^ ^]^
Gold NRs	Sub‐100 nm NR placed on DNA origami of ≈100 nm each side	PA imaging	Mice	4T1‐fLuc mouse breast‐cancer cells	DNA‐coated gold NRs were found to be more effective than uncoated ones	^[^ ^273^ ^]^
Nano bipyramids	Length 117 nm and width ≈36 nm	Raman SERS	Mice	Human breast cancer cells MCF‐7	Folic acid‐targeting employed	^[^ ^253^ ^]^

## 5 Translational Aspects of Gold Nanoclusters as a Theranostic Agent

Cancer nanotheranostics represents a major recent advancement in diagnosing and treating cancer and is now considered a paradigm shift in the field of experimental medicine. Several gold nanostructures have shown tremendous effects in animal models for Raman scattering and plasmonic photothermal therapy. One distinctive feature of AuNPs in cancer theranostics is the improvement of the efficacy with minimal toxicities. Another feature is enabling noninvasive imaging of diseased tissues by traceable NPs. Despite their high success in experimental models, studies of durable/long‐term clinical responses are very limited. Therefore, there is a need to establish the efficacy and excretion pathways of AuNPs in longer term models and eventually human trails. It would be of great value to bioengineer a method that can predict treatment response and that will enable optimization of the likelihood of therapeutic success and reduce the risks and expense of unnecessary treatment.

AuNPs are now routinely used within in vitro devices, as diagnostic tools in the clinic: such as pregnancy test kits containing antibody‐conjugated AuNPs, which are applied for the colorimetric detection of human chorionic gonadotropin in urine and allowing a naked‐eye readout. AuNPs undergo plasmonic colorimetric change on detecting a change in hormone levels of urine. A wide range of NP‐based detection methods, including biomarkers in blood, urine, and saliva, are also in clinical use.

AuNPs are in the translation phase to in vivo application within a number of labs around the world. In an ongoing clinical trial, Naomi Halas from the Rice University and Steven Canfield from the McGovern Medical School at University of Texas are using AuNPs to treat prostate cancer.^[^ ^275^ ^]^ Their recent findings so far are promising, and side effects are relatively minimal.^[^ ^276^ ^]^ They have used gold–silica nanoshells (with a diameter of 150 nm, 8.28 µg g^−1^ (range, 1.15–33.12 µg g^−1^) of concentration of NPs and dose of 7.5 mL kg^−1^) used in this procedure comprise of tiny layers of silica glass in spherical shapes with a thin layer of gold coating on each sphere. These NPs have successfully reached target sites and been stimulated by laser light (NIR (810 ± 10 nm) was delivered continuously for 3 min at a power level subablative in the absence of NPs. Laser power was delivered via a dual lumen, water‐cooled catheter), which kills the cancer cells selectively. These core–shell NPs were cleared through the liver, while some remain sequestered in the liver and spleen with no noticeable side effects. 16 men aged 58–79 with low‐ to intermediate‐grade prostate cancer were involved in this clinical trial. The treatment was successful in 87.5% of lesions treated at 1 year of follow‐up. This clinical work may be able to address unanswered questions for the clinical translation of gold nanotechnology coupled with optical diagnostics and treatment procedures.

Numerous preclinical studies have shown the potential of AuNPs for the diagnosis and treatment of cancer, but the clinical translation of AuNP in the diagnosis/treatment modalities is still in its infancy, since long‐term chronic toxic impact of NPs, off‐target toxicity, metabolism, neurotoxicity, immunotoxicity, fate of protein‐based nanoformulations, clearance/excretion mechanism, lack of specific target knowledge, disease detection capabilities, and the use of diagnosis to assist or guide NPs‐based therapy procedures remains unexplored.

There is a lot of controversy on the localization, toxicity, biocompatibility, circulation, biodistribution, and clearance of NPs in literature to date: all these parameters give conflicting results based on the diversity of experimental protocols (such as size, shape, functionalization method, animal model, administration route). However, these theranostic procedures may need to be standardized in meeting product quality assessments, NPs characterization, safety evaluations, and experimental protocols of the in vitro and in vivo work.^[^ ^17^, ^277^, ^278^, ^279^ ^]^ Significant variations noted among individual tumors and therapeutic responses from one patient to another and temperature patterns between individuals are critical in introducing new approaches and procedures in clinical settings. These make it hard to interpret the results of small studies and that more statistical power or robust protocols are needed to understand these variations. Therefore, the most important translational barriers need to be considered during laboratory experiments to make the technology safe and effective by determining the selection, as well as the delivery conundrum of right diagnostic/treatment option, right cell, and right dose, with minimal collateral damages.

Specifically, the translation of photothermal theranostics could be challenging because of the considerable variations of temperature in tumors, the magnitude and duration of temperature applied, nonuniform distribution of the temperature within diseased tissue, cooling effects of surrounding tissues/blood vessels, location of the tumor, distance from the light source, density of the tumor, vascularization of the tissue, and other uncontrollable factors. Therefore, a standardized experimental procedure should be established to enable comparative evaluations of each approach. The use of the newly developed T‐SESORS/T‐SORS approaches outlined above make a direct noninvasive measure of the temperature using the Raman signal induced by the PTT laser. This could have a profound effect on clinical utility and precision of this approach.

Furthermore, the scale‐up of cost‐effective, reproducible AuNP production, including their complex geometries/morphologies and multilayered biocojugated is another translational barrier. However, research advances in this field are currently being made by numerous academic researchers outlined here. Therefore, proposed programmes of work should be aligned with clinical endpoints. This requires better coordination between academic researchers and clinicians/clinical investigators. With the transition of AuNPs from the benchtop to the bedside, we need to address unanswerable clinical questions about the fundamental problems in translating this “golden age” technology into the “postmodern age.”

## 6 Conclusions and Future Perspectives

There has been a growing trend in developing various novel engineered AuNPs for integrating both optical diagnostics and photothermal therapy within a single procedure. Advancing gold nanostructure‐based Raman nanotheranostics may be pivotal for translating from in vitro therapeutics and detection to more complex biological systems in vivo within clinical settings.

Furthermore, there have been major recent advances in surface enhanced spatially offset Raman spectroscopy (SESORS) to provide optical imaging and lesion localization combined with PTT and direct optical feedback to provide accurate in vivo temperature and pH monitoring. We have reviewed evidence for this growing consensus and have highlighted the combination of two modalities (diagnosis and treatment) in a single, efficient, cost‐effective, and targeted procedure. This review also provides significant advancements by addressing the challenges to finding new techniques of functionalizing AuNPs with molecules that can facilitate effective binding, formation of a corona, clearance, biocompatibility, biodistribution, and toxicity. It has also addressed the unmet clinical need and translational barrier of immediate and effective nonsurgical cancer detection and diagnosis with high specificity and sensitivity using gold nanostructures in a single, effective, nonsurgical procedure. Gold nanostructures have widely been explored for medicinal applications but still there is a long delay in the translation of these (NPs) to be commercialized for routine in vivo light‐triggered treatment options.

Raman‐based multiplexed theranostic systems can elucidate the complexity of disease function and provide for linked diagnostic and therapeutic procedures. Raman scattering may revolutionize theranostic procedures including monitoring of trafficking at cellular/subcellular levels, cellular localization, reassessment of EPR, and may provide key insights into transcytosis, exosomal transport, and hyperthermia control in achieving clinically‐relevant diagnostic aided and image‐based therapeutic responses.

Many new NPs have been developed and tested for cancer theranostics with overall therapeutic success rates in cell culture and animal models remaining very high. However, there are many barriers in translating and adopting them into clinical settings such as: i) limited understanding of long‐term toxic effects of AuNPs, ii) nonspecific biodistribution profiles, short plasma circulation time, and rapid systemic elimination, iii) relatively low accumulation at the target site, and iv) fate of NPs in the bloodstream of a living system and in the cytoplasm of the cell, as well as in the media in which cells grow (a mixture of electrolytes, proteins, nutrients, and metabolites). In these regards, Raman spectroscopic approaches could potentially play an important role in detecting nano–bio interactions at cellular/subcellular levels. There is, however, considerable room for further basic science advancing the understanding of biological interactions with AuNP on the nanoscale, novel NP engineering, Raman signal recovery, NP localization, dosimetry, and real‐time monitoring of hyperthermia, all requiring a concerted effort for successful translation and adoption of clinical tools utilizing Raman nanotheranostics.

## Conflict of Interest

The authors declare no conflict of interest.
